# Cullin‐associated and neddylation‐dissociated 1 regulate reprogramming of lipid metabolism through SKP1‐Cullin‐1‐F‐box^FBXO11^‐mediated heterogeneous nuclear ribonucleoprotein A2/B1 ubiquitination and promote hepatocellular carcinoma

**DOI:** 10.1002/ctm2.1443

**Published:** 2023-10-14

**Authors:** Hao Zhang, Peng Xia, Zhangshuo Yang, Jie Liu, Yimin Zhu, Zan Huang, Zhonglin Zhang, Yufeng Yuan

**Affiliations:** ^1^ Department of Hepatobiliary and Pancreatic Surgery Zhongnan Hospital of Wuhan University Wuhan China; ^2^ Clinical Medicine Research Center for Minimally Invasive Procedure of Hepatobiliary & Pancreatic Diseases of Hubei Province Wuhan China; ^3^ TaiKang Center for Life and Medical Sciences Wuhan University Wuhan China; ^4^ College of Life Sciences Wuhan University Wuhan China; ^5^ Department of Organ Transplantation Qilu Hospital of Shandong University Jinan China; ^6^ Department of Breast Surgery Affiliated Hospital of Hebei University Baoding China

**Keywords:** adeno‐associated virus, hepatocellular carcinoma, lipid metabolism reprogramming, SCF complex, ubiquitination

## Abstract

**Background:**

Enhanced de novo lipogenesis is essential for hepatocellular carcinoma (HCC). Abnormally high cullin‐associated and neddylation‐dissociated 1 (CAND1) expression is associated with poor clinical prognosis in HCC. The SKP1‐Cullin‐1‐F‐box (SCF) complex consists of the SKP1, Cullin‐1 and F‐box proteins (FBPs) and performs multiple functions including adipogenesis. SCF complex was modulated by CAND1, but Whether and how the CAND1 promotes HCC by regulating SCF complex and lipogenesis are unknown.

**Methods:**

HCC samples were used to analyze the correlations between CAND1 expression and clinicopathological characteristics such as survival and prognosis. The in vitro functions of CAND1, FBXO11 and heterogeneous nuclear ribonucleoprotein A2/B1 (hnRNPA2B1) were measured by cell proliferation, colony formation and migration assays. The in vivo functions were tested in multiple mouse liver cancer models including patient‐derived xenograft (PDX), cell line‐derived xenograft and AKT/NRASV12‐induced primary liver cancer models. Injections of adeno‐associated virus targeting CAND1 (AAV‐shCAND1) were performed to evaluate the therapeutic efficacy of targeting CAND1. RNA‐Seq and lipidomic assays followed by serial biochemical experiments including mass spectrometry, immunoprecipitation and GST pull‐down were performed to dissect the underlying mechanisms.

**Results:**

CAND1 promoted the expression of lipid synthesis genes by disrupting SCF complex assembly and lipid accumulation. Furthermore, we identified hnRNPA2B1 as a novel F‐box protein 11 (FBXO11)‐binding partner. FBXO11 directly bound to hnRNPA2B1 and promoted hnRNPA2B1 ubiquitination and subsequent degradation. Our evaluations of the therapeutic efficacy of AAV‐shCAND1 injections confirmed that targeting the CAND1‐SCF^FBXO11^‐hnRNPA2B1A signalling axis was therapeutically effective. CAND1 downregulation significantly reduced the tumour burden in a primary mouse liver cancer model and a PDX model.

**Conclusions:**

Our results highlight that CAND1 is associated with poor prognosis in HCC and regulates lipid metabolic reprogramming by dissociating the SCF complex. Targeting the CAND1‐SCF^FBXO11^‐hnRNPA2B1 axis may be a novel strategy for HCC treatment.

## Graphical abstract

Our results identify cullin‐associated and neddylation‐dissociated 1 (CAND1) as an oncogene that regulates lipid metabolic reprogramming through dissociating the Skp1‐Cul‐FBXO11 complex. Targeting CAND1‐SCF(FBXO11)‐hnRNPA2B1 axis may be a novel strategy against hepatocellular carcinoma.

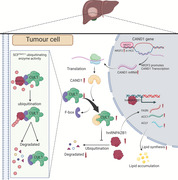

## 1 INTRODUCTION

Hepatocellular carcinoma (HCC) is characterized by multiple features of metabolic reprogramming, one of which is enhanced de novo lipogenesis.^1^, ^2^ Enhanced de novo lipogenesis is characterized by increased activity and expression of various lipogenic enzymes in HCC.^3^, ^4^ However, the underlying mechanisms remain to be addressed.

Metabolic dysregulation plays a pivotal role in tumorigenesis and development, and increasing evidence indicates that lipid metabolism is essentially reprogrammed in tumours.^5^, ^6^ Lipids are reported to be an important energy source to fuel tumour progression and metastasis.^7^ In addition, studies have confirmed lipid droplets (LDs) and lipogenesis play an important role in the development and stemness of HCC.^8^, ^9^, ^10^, ^11^

SKP‐Cullin1‐F‐box (SCF) complexes have been suggested to play important roles in multiple processes including adipogenesis and lipogenesis.^12^, ^13^ The typical biological function of the SCF complex is to induce the degradation of various substrates through ubiquitination. Different F‐box proteins (FBPs) determine distinct SCF complexes that recognize a variety of substrates^14^, ^15^, ^16^ and have multiple pathophysiological functions.^17^, ^18^ Whether SCF complexes contribute to hepatocarcinogenesis by regulating lipogenesis is not known.

Cullin‐associated and neddylation‐dissociated 1 (CAND1) regulates the dissociation and assembly of SCF complexes.^19^, ^20^, ^21^ Accumulative evidence demonstrates that CAND1 is upregulated in various cancers and regulates lipogenesis.^22^, ^23^ In addition, high expression of CAND1 plays pro‐oncogenic roles in a variety of cancers.^24^, ^25^ HCC data from The Cancer Genome Atlas (TCGA) suggest that CAND1 mRNA is upregulated and an indicator of an unfavourable prognosis. However, whether and how CAND1 affects hepatocarcinogenesis by regulating SCF complexes and lipogenesis remains unknown. Gene therapy is defined as the introduction of genetic material into a target cell for therapeutic benefit.

Gene delivery using viral vectors is one potential therapeutic option. Adeno‐associated virus (AAV) is recognized as a promising vector for gene therapy applications because it is nonpathogenic, replication‐defective and capable of long‐term transgene expression.^26^, ^27^ As a result of these properties, AAV vectors have enabled clinical successes in several recent clinical trials.^28^, ^29^ In the current study, we confirmed CAND1 as a promising therapeutic target for AAV treatment in HCC.

Specifically, we identify CAND1 as a negative regulator of the SCF^FBXO11^ complex in HCC and present results showing that the disassembly of the SCF^FBXO11^ complex stabilizes heterogeneous nuclear ribonucleoprotein A2/B1 (hnRNPA2B1), which enhances lipogenesis to promote HCC in vitro and in vivo. In vivo studies are designed to evaluate the antitumor efficacy of AAV‐mediated gene therapy and we show that targeting CAND1 has a therapeutic effect on HCC in cell line‐derived and patient‐derived xenograft (CDX and PDX) models. We also identify and validate the therapeutic effect of AAV‐based targeting of the CAND1‐SCF^FBXO11^‐hnRNPA2B1 signalling pathway on HCC. Together, our findings provide novel insights into the mechanisms governing the reprogramming of lipid metabolism and suggest new strategies against HCC.

## 2 MATERIALS AND METHODS

### 2.1 Clinical and tumour specimens

Human HCC tissues and paired adjacent non‐tumour tissues (74 pairs in total) were collected from Zhongnan Hospital of Wuhan University. The adjacent non‐tumour liver tissue specimens were obtained 5–7 cm away from the edge of the tumour. Then, HE staining was performed to distinguish tumour and adjacent non‐tumour tissue specimens. Consent from each patient was obtained before the processing of the samples. Patients with preoperative anticancer therapy, distant metastasis, and loss of follow‐up were excluded from this study. All experiments involving human tissue samples were performed in accordance with relevant guidelines and regulations approved by the Medical Ethics Committee of Zhongnan Hospital of Wuhan University (Scientific Ethical Approval No. 2020100). All patients signed informed consent forms before the experiments.

### 2.2 CDX model

All mice were housed in an SPF environment. All animal experiments were carried out in accordance with the National Research Council's Guide for the Care and Use of Laboratory Animals. Animal experiments were approved by the Experimental Animal Welfare Ethics Committee of Zhongnan Hospital of Wuhan University (Approval No. ZN2022005).

To establish the subcutaneous tumour formation mouse model, 6‐week‐old male BALB/c‐nu mice were injected subcutaneously on the bilateral flanks with 3×106 Huh‐7 cells suspended in 200 μL of culture medium. Tumour volumes were calculated as (π/6) × length × width^2^. Two weeks later, the mice were euthanized by cervical dislocation after receiving isoflurane anaesthesia. Tumours were harvested and weighed.

### 2.3 Lung metastasis and liver metastasis models

Experimental lung metastases were identified following a previously described method.^30^ Briefly, differentially treated Huh7 cells in the logarithmic growth phase were injected into 6‐week‐old male nude mice through the tail vein. The nude mice were sacrificed one month after injection, and the lung tissues were collected. Experimental liver metastases were generated by intrasplenic/portal injections of 2×10^5^ Huh‐7 cells, followed by splenectomy. Mice were sacrificed 28 days post‐surgery, and metastatic foci were observed.

### 2.4 Primary liver tumour model

Sleeping Beauty transposon (SB) vectors overexpressing AKT (pKT2/CLP‐AKT) and NRASV12 (pT/Caggs‐NRASV12) oncogenes were used to induce HCC in 4‐week‐old male mice.^31^ Transposon vectors and transient expression vectors carrying Sleeping Beauty 100 (SB100) transposase were solubilized in 0.9% NaCl solution. Oncogenes (12.5 μg of each oncogene plasmid with 2 μg of the SB100 plasmid) were introduced by hydrodynamic injection through the tail vein within 8 s. The total volume constituted 10% of the mouse's body weight. On days 3 and 17 after the hydrodynamic transfection, we injected the mice through the tail vein with a control lentivirus or a shCAND1‐expressing lentivirus. One month after hydrodynamic transfection, mice were euthanized, and mouse livers were harvested immediately.

### 2.5 PDX model

For the PDX mouse model, mice were implanted with a tumour obtained from a patient with HCC. After the tumours reached approximately 80–120 mm^3^, the mice were randomly assigned to groups with 5 mice per group. shCAND1 (50 mg/kg) or phosphate‐buffered saline (PBS) (25 mg/kg) was intratumorally injected every other day (four injections in total), and the tumour volume was measured every 2 days. The tumours were resected from sacrificed mice for weighing and immunofluorescence staining. For PDX survival analysis, PDX tumour fragments were subcutaneously inoculated into the dorsal flank of nude mice. When the tumours reached 80–120 mm^3^, the mice were assigned randomly to groups and treated as described above. The experimental endpoint was defined as either death or a tumour size greater than 1500 mm^3^. Mice with tumours larger than 1500 mm^3^ were euthanized.

Tumour interstitial fluid pressure (IFP) evaluation was conducted using the wick‐in‐needle technique before mice were sacrificed. When the mice were anaesthetized, a 23‐gauge needle with a 2–3 mm side hole was inserted into the centre of the tumour and IFP was recorded when the value remained stable.

### 2.6 Recombinant AAV‐shRNA virus construction

AAV serotype 8 (AAV8) capsid was chosen for in vivo studies due to its strong liver tropism. Recombinant adeno‐associated virus vectors based on AAV8 have been previously developed for liver‐targeted gene therapy of numerous genetic diseases. For in vivo studies, the CAND1 shRNA sequence was packaged into the AAV8 virus obtained from Tsingke. The construct was transfected into HEK‐293 cells, generating CAND1 shRNA AAV(AAV‐shCAND1). Viral vectors were screened to be free from replication‐competent viruses, lipopolysaccharides and bacteriological contaminants. The homologous AAV8 empty virus (AAV‐shNC) was used as a blank control. In the primary liver tumour model, mice were injected via the tail vein with AAV‐shCAND1 or AAV‐shNC. In the PDX model, intratumoral injection of AAV‐shCAND1 or AAV‐shNC (100 μL, 4 × 10^11^ vp/mL) was conducted.

### 2.7 Cell culture

HCC cell lines, including the Hep3B, Huh7, HCCLM3, HepG2, Hep 1−6 and PLC5 cell lines and the HEK293T cell line, were purchased from Shanghai Cell Bank of the Chinese Academy of Science. The normal human hepatic cell line THLE‐3 was purchased from the American Type Culture Collection (ATCC). Cell line authentication was performed by the ATCC Human STR Profiling Cell Authentication Service. Hep3B cells were cultured in MEM (HyClone) supplemented with 10% fetal bovine serum (FBS) purchased from Gibco. The other cells were maintained in DMEM (HyClone) supplemented with 10% FBS (Gibco). All cells were cultured at 37°C in a 5% CO_2_ incubator.

### 2.8 RNA preparation and quantitative reverse transcription polymerase chain reaction

RNA was extracted using the standard TRIzol (Invitrogen) RNA extraction method. After extraction, the Fast Quant RT Kit (Vazyme Biotech) was used for reverse transcription of the RNA into cDNA. Quantitative reverse transcription polymerase chain reaction (qRT‒PCR) was performed with an ABI 7300 real‐time PCR system (Applied Biosystems) using the SYBR‐Green (Vazyme Biotech) method. The following CAND1 primers were used: forward 5′‐ TGGATGCTGATGGTGGTGAT −3′ and reverse 5′‐ TCATGCCTTGTGCTAACTACAG −3′. The following β‐actin control primers were used: forward 5′‐ TGCGTGACATTAAGGAGAAG −3′, and reverse 5′‐ GCTCGTAGCTCTTCTCCA −3′. The results were validated with three independent experiments.

### 2.9 Western blot analysis

Western blotting was performed as previously described.^17^ Antibodies against FBXO11 (27610‐1‐AP), CUL1 (12895‐1‐AP), FBXO5 (EPR15320‐103), FBW7 (28424‐1‐AP), FBX15 (13024‐1‐AP), hnRNPA2B1 (14813‐1‐AP), Flag (80010‐1‐RR), HA (51064‐2‐AP), His (66005‐1‐Ig), FASN (10624‐2‐AP), ACC (21923‐1‐AP), PPARD (60193‐1‐Ig), PPARG (66936‐1‐Ig), NR2F2 (24573‐1‐AP), TNFR1 (21574‐1‐AP), TRAF2 (26846‐1‐AP), P65 (80979‐1‐RR), Caspase8 (66093‐1‐Ig) and β‐ actin (66009‐1‐Ig) were purchased from Proteintech. Antibodies against CAND1 (ab183748), PPARA (ab126285) and FBXL3 (ab96645) were purchased from Abcam. Antibodies against CAND2 (PA5‐43591) were purchased from ThermoFisher. Antibodies against FBXL12 (NB100‐1295; Novus Biologicals), FBXO44 (sc‐398020; Santa Cruz Biotechnology) and FBX18 (sc‐81563; Santa Cruz Biotechnology) were also purchased.

### 2.10 Cell proliferation assay

Cell proliferation was measured by means of Cell Counting Kit‐8 (CCK‐8) (C0038; Beyotime) and clonogenic assays. Briefly, cells were seeded at 2000 cells/well in 96‐well plates and cultured for 4 h. After the cells had adhered to the well, 10 μL of CCK‐8 working buffer was added to each well of the 96‐well plate, and the cells were incubated at 37°C for 1 h. The absorbance at 450 nm in each well was then measured. For clonogenic assays, cells were seeded at 1000 cells/well in 6‐well plates. After incubation for 2 weeks, the cells were washed with PBS, fixed, and stained with crystal violet. Colony numbers were counted with the Cell Counter ImageJ plugin.

### 2.11 Transwell and scratch wound assays

Transwell experiments were performed as described.^30^ Briefly, 2×10^4^ cells in 100 μL of serum‐free medium were added to the upper Transwell insert chamber, and 500 μL of culture medium with 20% FBS was added to the lower insert well. After 24 h in culture, the cells on the upper surface of the membrane were removed, and the cells that had migrated to the lower surface were fixed with 4% formaldehyde and then stained with 0.05% crystal violet. Cells for the scratch assay were grown to confluency, and the wound was generated with a pipette tip. Images were taken immediately after the scratch was made and subsequently at 24‐h intervals using an inverted microscope.

### 2.12 Coimmunoprecipitation and ubiquitination assays

The Coimmunoprecipitation (co‐IP) assay was performed with a Pierce Crosslink Magnetic IP/Co‐IP kit (Thermo Fisher Scientific) according to the manufacturer's instructions. Whole‐cell lysates were incubated with the desired antibodies, and a target protein was pulled down with protein A/G magnetic beads. The microbeads were washed with a low‐concentration binding buffer, which caused the bound protein to dissociate from the antibody‐conjugated beads. The eluate was collected and analyzed by western blotting and MS.

For the ubiquitination assay, cells cotransfected with the indicated plasmids were lysed in cold IP lysis buffer containing 1% sodium dodecyl sulfate. For MG132 treatment, a 10 mM stock solution was made by dissolving MG132 (Solarbio) in DMSO, and the final MG132 concentration was 10 μM. Cells were treated with MG132 at 6 h before harvesting the cells. Subsequently, the lysates were diluted tenfold with IP lysis buffer and subjected to ultrasonic disruption and subsequent centrifugation. The next steps were the same as those used for the co‐IP assay.

### 2.13 GST pull‐down assay

A GST pull‐down assay was performed using a Magne GST pull‐down system (Promega) according to the manufacturer's instructions. Briefly, proteins were expressed in Escherichia coli BL21 (DE3) overnight at 18°C, and purification of GST fusion proteins was performed with GST‐tag purification resin (P2253; Beyotime). Total cell lysates were subjected to GST pull‐down by magnetic beads overnight at 4 °C (P2138; Beyotime) with immobilized GST‐control or GST‐tagged proteins as indicated. The beads were then washed three times with chilled lysis buffer. Eluted samples and inputs were subjected to western blotting.

### 2.14 Detection of LDs, intracellular triglyceride and cholesterol

LDs were stained with a BODIPY 493/503 probe (25892‐10; AmyJet Scientific). After cell plating on glass coverslips and fixation, the cells were washed and incubated with 1 μg/mL BODIPY 493/503 for 10 min at room temperature. The number and area of the BODIPY dots per cell in at least 100 cells per independent experiment were determined with ImageJ software. Lipid accumulation in tumour tissue was visualized by oil‐red O staining. Tissue sections were fixed in 2% paraformaldehyde and stained with oil red O (C0158S; Beyotime) for 30 min at room temperature. Sections were washed with 60% isopropanol and water before imaging. Intracellular triglyceride (TG) and total cholesterol (TC) contents were quantified with a TG assay kit (ab65336; Abcam) and a TC assay kit (ab65359; Abcam) according to the manufacturer's instructions.

### 2.15 Statistical analysis

All numerical data are presented as the mean   ± standard deviation based on at least three experiments unless otherwise indicated. The chi‐square test was used for categorical variables. Data graphics and statistical analysis were performed using Prism 5 (GraphPad Prism). Two‐tailed Student's t‐tests were performed to determine significant differences obtained through in vitro and in vivo experiments. Assays were run in triplicate and repeated at least thrice. The data analysts were blinded to the group assignment. *p* < .05 was considered to indicate a statistically significant difference. Significance was defined as *p* < .05(*), *p* < .01(**) or *p* < .001(***).

## 3 RESULTS

### 3.1 Abnormally high CAND1 expression is associated with poor clinical prognosis in HCC

**FIGURE 1:**
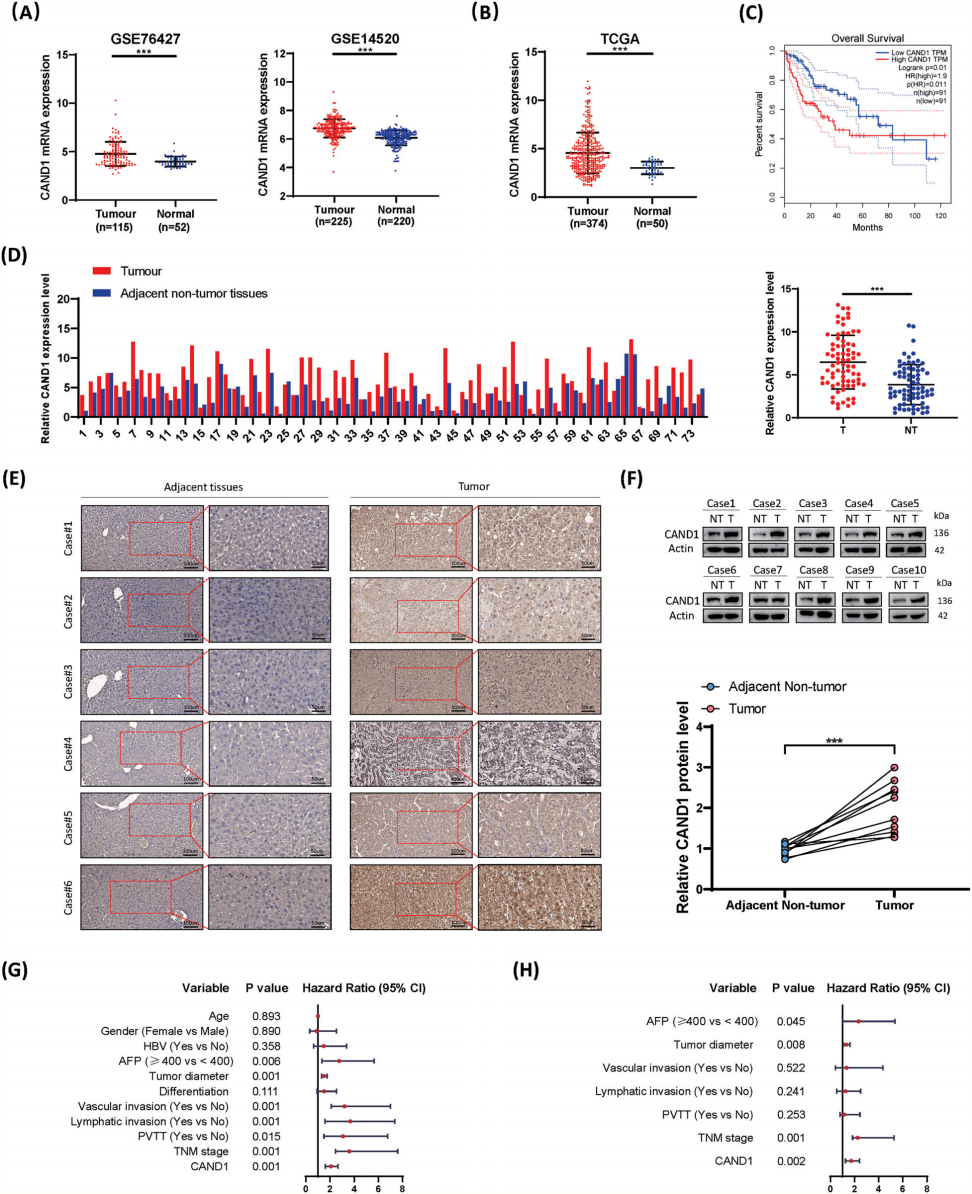
Abnormally high cullin‐associated and neddylation‐dissociated 1 (CAND1) expression is associated with poor clinical prognosis in hepatocellular carcinoma (HCC). (A) CAND1 mRNA is highly expressed in HCC tumours in the GEO database. (B) CAND1 mRNA is highly expressed in HCC tumours in the Cancer Genome Atlas (TCGA) database. (C) TCGA data show a significant correlation between CAND1 expression and overall survival. (D) CAND1 expression is increased in tumour tissues (T) compared to the paired non‐tumour tissues (NT) (*n* = 74) obtained from Zhongnan Hospital (The adjacent normal liver tissue specimens were obtained 5–7 cm away from the edge of the tumour). (E) Representative immunohistochemical staining of CAND1 in HCC tumour and NT tissues. (F) CAND1 expression was detected by western blotting. (G) Results of the univariate Cox regression analysis. (H) Multivariate Cox regression analysis confirms the independence of CAND1 as a predictive factor. *p* < .05(*), *p* < .01(**) or *p* < .001(***).

Multiple HCC datasets (GSE76427 and GSE14520 datasets from the Gene Expression Omnibus (GEO) database and HCC data from TCGA) demonstrated that CAND1 was highly expressed in HCC and tightly associated with poor prognosis (Figure 1A–C, *p* < .001). Moreover, we confirmed CAND1 upregulation at the mRNA and protein levels in our patient cohort (Figure 1D–F). Furthermore, we observed that the expression level of CAND1 was positively associated with tumour diameter, vascular invasion, and TNM stage in HCC patients (Table 1). Univariate Cox regression analysis revealed that high expression of CAND1 was associated with poor prognosis (Figure 1G). Multivariate Cox regression analysis confirmed that CAND1 and TNM stage were independent prognostic factors (Figure 1H and Table 2). We further explored why CAND1 is up‐regulated in HCC. Through transcription factor prediction, it was found that nuclear receptor subfamily 2 group F member 2 (NR2F2) may be one of the transcription factors regulating CAND1 expression (Figure S1A). Results demonstrated that NR2F2 upregulated the CAND1 expression by binding to its promoter region (Figure S1B–G). Overall, these observations demonstrate that CAND1 overexpression is tightly associated with HCC and may serve as an unfavourable prognostic factor.

**TABLE 1:** Correlation between cullin‐associated and neddylation‐dissociated 1 (CAND1) expression and clinicopathologic characteristics of hepatocellular carcinoma patients.

	CAND1		
Characteristics	Low	High	*p*/χ2‐Value
Age (years)	54.78 ± 10.512	53.73 ± 11.019	0.675
Sex			0.496
Male	31	33	
Female	6	4	
HBV infection			0.425
Yes	26	29	
No	11	8	
AFP			0.485
≥400	20	17	
<400	17	20	
Tumour diameter (cm)	4.06 ± 2.264	5.30 ± 2.097	0.017
Vascular invasion			0.043
Yes	4	11	
No	33	26	
TNM stage			0.041
I	12	7	
II	20	14	
III	3	11	
IV	2	5	
PVTT			0.394
Yes	2	4	
No	35	33	
Lymphatic invasion			0.233
Yes	2	5	
No	35	32	
Differentiation			0.632
Poorly	10	8	
Moderately	20	24	
Well	7	5	
CAND1	1.44 ± 0.364	3.37 ± 1.095	<0.001

Abbreviations: AFP, alpha‐fetoprotein; HBV, hepatitis B virus; PVTT, portal vein tumour thrombosis.

**TABLE 2:** Univariate and multivariate Cox‐regression analysis of various prognostic parameters in patients with liver cancer.

	Univariate analysis		Multivariate analysis	
	*p*	HR (95% CI)	*p*	HR (95% CI)
Age	0.893	0.998 (0.967–1.029)		
Sex (Female vs. Male)	0.890	0.884 (0.314–2.524)		
HBV (Yes vs. No)	0.358	1.473 (0.645–3.366)		
AFP (≥400 vs. < 400)	0.006	2.749 (1.339–5.644)	0.045	2.336 (1.020–5.352)
Diameter	0.001	1.508 (1.295–1.757)	0.008	1.319 (1.073–1.621)
Vascular invasion (Yes vs. No)	0.001	3.188 (2.099–6.996)	0.522	1.345 (0.415–4.361)
Lymphatic invasion (Yes vs No)	0.001	3.679 (1.583–7.363)	0.241	1.255 (0.526–2.499)
PVTT (Yes vs. No)	0.015	3.084 (1.493–6.779)	0.253	1.132 (0.792–2.450)
TNM	0.001	3.596 (2.453–7.601)	0.001	2.262 (1.834–5.262)
Differentiation	0.111	1.518 (0.909–2.535)		
CAND1	0.001	2.069 (1.609–2.662)	0.002	1.736 (1.245–2.420)

Abbreviations: CI, confidence interval; HR, hazard ratio.

### 3.2 CAND1 functions as an oncogene in HCC in vitro and in vivo

**FIGURE 2:**
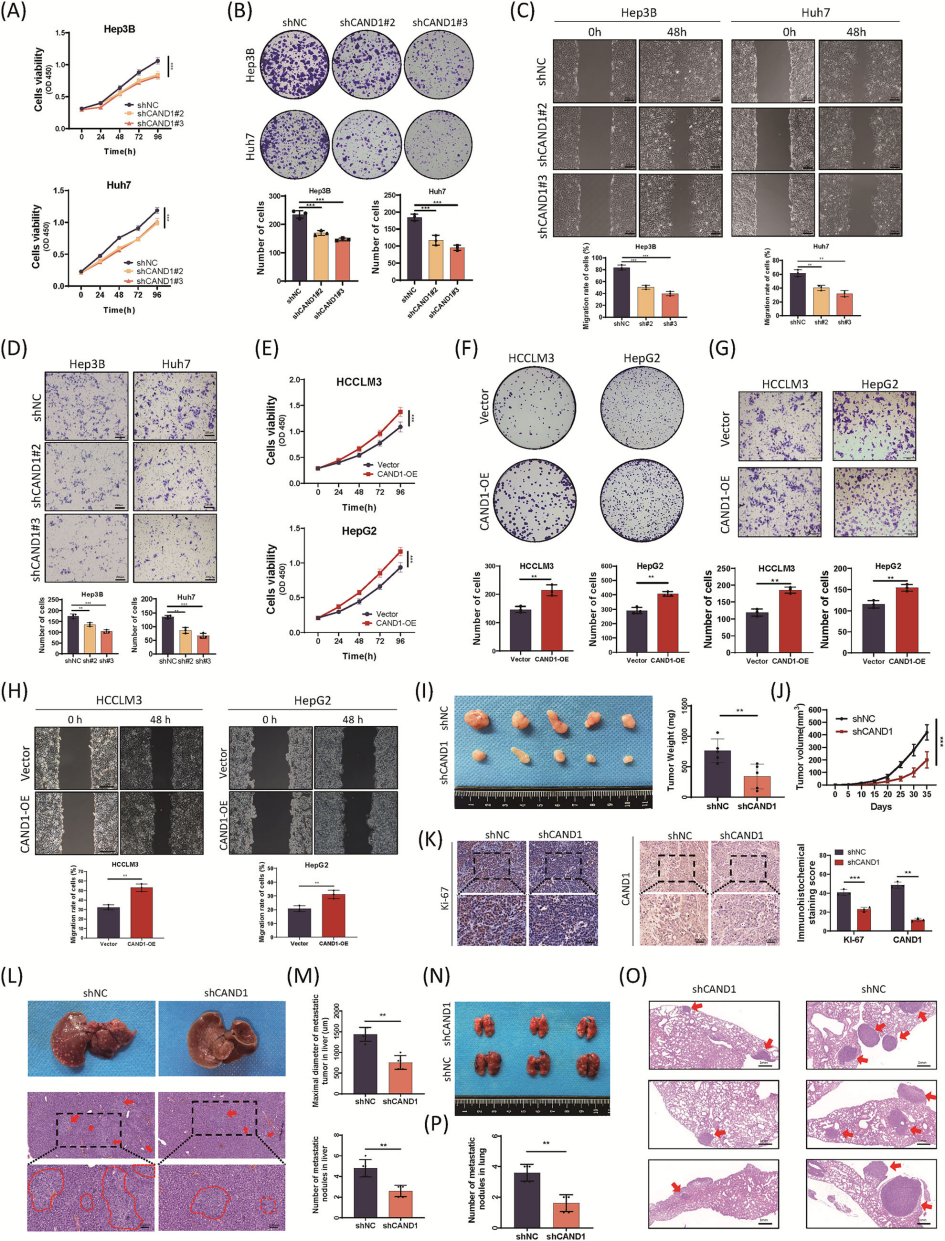
Cullin‐associated and neddylation‐dissociated 1 (CAND1) functions as an oncogene in hepatocellular carcinoma (HCC) in vitro and in vivo. (A) Cell viability was measured by Cell Counting Kit‐8 (CCK‐8) assay. (B) CAND1 knockdown impairs colony formation. (C) Wound healing assays show reduced cell migration in CAND1 knockdown cells. (D) Transwell experiments confirmed that CAND1 knockdown suppresses cell invasion. (E) CAND1 overexpression significantly promotes cell growth, as detected by CCK‐8. (F) CAND1 overexpression increases colony formation. (G, H) Transwell and wound healing assays showed that CAND1 overexpression promotes cell invasion and migration. (I) Image of subcutaneous xenograft tumours and the statistical analysis of tumour weights. (J) Growth curve of xenografts in the shCAND1 and control groups. (K) Immunohistochemical analysis of xenografts shows a decrease in Ki‐67 in the CAND1 knockdown group. (L) Typical gross appearance and hematoxylin and eosin (H&E) staining of liver metastatic tissue. (M) Maximal tumour diameters and number of liver metastasis nodules under light microscopy. (N) Representative images of pulmonary metastatic models. (O) H&E staining of metastatic nodules in the lungs. (P) The number of lung metastatic nodules was monitored under a dissecting microscope. All cellular experiments were run in triplicate and repeated three times. *p* < .05(*), *p* < .01(**) or *p* < .001(***).

CAND1 protein expression was relatively high in the Hep3B and Huh7 cell lines and relatively low in HCCLM3 and HepG2 cells (Figure S2A). Therefore, we knocked down CAND1 in Hep3B and Huh7 cells and overexpressed CAND1 in HCCLM3 and HepG2 cells (Figure S2B,C). CAND1 knockdown impaired the proliferation, colony formation, migration and invasion of Hep3B and Huh7 cells (Figure 2A–D). In contrast, CAND1 overexpression caused the opposite phenotypes in HCCLM3 and HepG2 cells (Figure 2E–H). We further tested the in vivo function of CAND1 in xenograft mouse liver cancer models. CAND1 knockdown significantly delayed Hep3B cells from forming subcutaneous tumours in nude mice and CAND1 knockdown tumours exhibited reduced Ki‐67 staining compared to shNC tumours (Figure 2I–K). Furthermore, intrasplenic injection of CAND1 knockdown Hep3B cells led to fewer liver tumour nodules than injection of shNC cells, while tail vein injection of CAND1 knockdown Hep3B cells caused significantly fewer tumour nodules in the lung than injection of shNC cells (Figure 2L–P). Overall, these results demonstrate that CAND1 promotes HCC cell proliferation, colony formation, migration, and invasion in vitro and that CAND1 deficiency impairs subcutaneous tumour formation and metastasis in vivo.

### 3.3 CAND1 promotes HCC by enhancing lipogenesis

**FIGURE 3:**
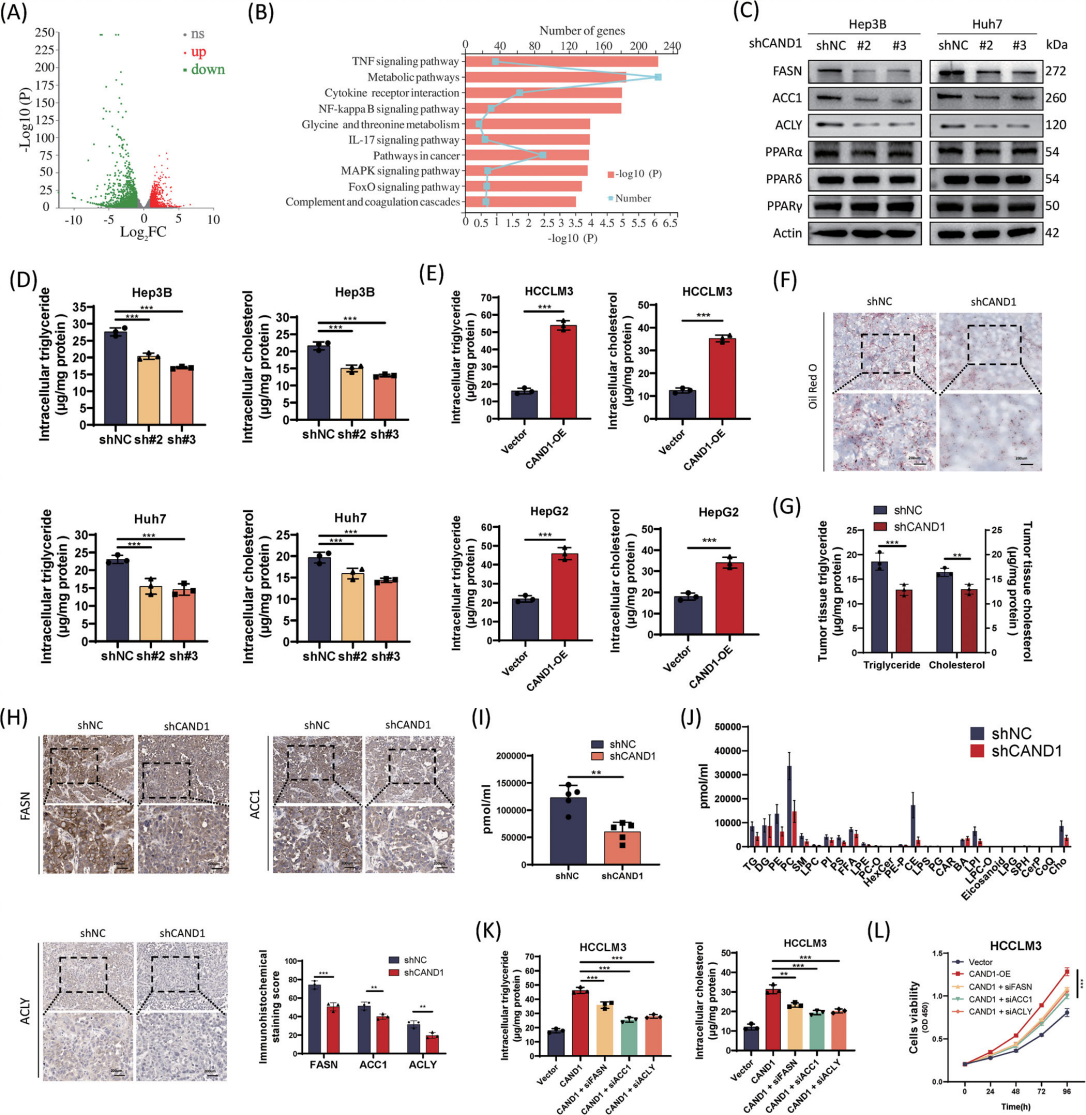
Cullin‐associated and neddylation‐dissociated 1 (CAND1) regulates hepatocellular carcinoma (HCC) lipid metabolism in vitro and in vivo. (A) Volcano plot showing differentially expressed genes in RNA‐Seq analysis. (B) Kyoto Encyclopedia of Genes and Genomes (KEGG) enrichment analysis showed enrichment of metabolic pathways. (C) Fatty acid synthesis and oxidation‐associated proteins were detected by Western blotting. (D) Intracellular triglyceride and cholesterol contents were measured in Hep3B and Huh7 cells expressing shNC, shCAND1#2, or shCAND1#3. (E) Cellular triglyceride and cholesterol contents were measured in HCCLM3 and HepG2 cells overexpressing CAND1. (F) Oil‐Red‐O staining of xenografts. (G) Tumour tissue triglyceride and cholesterol levels were measured in xenografts. (H) Immunohistochemical analysis of xenografts showed a decrease in FASN, ACC1 and ACLY in the CAND1 knockdown group. (I) Lipid levels in all classes in CAND1 knockdown and control cells. (J) Differences in total lipids in CAND1 knockdown cells. (K) The knockdown of FASN, ACC1 and ACLY reversed the intracellular triglyceride and cholesterol levels caused by CAND1 overexpression. (L) Cell Counting Kit‐8 (CCK‐8) assays showed that the knockdown of FASN, ACC1 and ACLY reversed the increase in cell viability caused by CAND1 overexpression. CAND1‐OE: CAND1 overexpression; LD: lipid droplets. All cellular experiments were run in triplicate and repeated three times. *p* < .05(*), *p* < .01(**) or *p* < .001(***).

To elucidate the underlying mechanisms, we performed RNA‐Seq analysis in control and CAND1‐knockdown Huh‐7 cells. A volcano plot revealed 2513 differentially expressed genes (DEGs), of which 1061 genes were upregulated and 1452 genes were downregulated in CAND1 knockdown cells (Figure 3A). Kyoto Encyclopedia of Genes and Genomes (KEGG) enrichment analysis of DEGs showed that metabolic pathways with the largest number of DEGs (233 genes) were among the top 10 pathways (*p* < .001) (Figure 3B). In particular, many of the DEGs enriched in metabolic pathways were involved in lipid metabolism. Major genes responsible for the synthesis of fatty acids, such as fatty acid synthase (FASN), acetyl‐CoA carboxylase 1(ACC1) and ATP citrate lyase (ACLY), were downregulated in the CAND1 knockdown group whereas fatty acid oxidation‐associated genes (PPARA, PPARD and PPARG) in CAND1‐knockdown cells were not affected (Figure 3C). Consistent with the gene expression pattern, significant decreases in the intracellular accumulation of TGs, cholesterol (CE) and LDs were observed in CAND1‐knockdown cells whereas CAND1 overexpression had the opposite effects (Figure 3D,E and Figure S3). Subcutaneous tumours derived from CAND1 knockdown cells in a xenograft nude mouse model also exhibited downregulation of FASN, ACC1 and ACLY, enhanced oil red O staining and an increase in TAG content (Figure 3F–H). Furthermore, quantitative lipidomic analysis showed that the total content of all classes of lipids in CAND1‐knockdown cells was significantly lower than that in shNC cells (Figure 3I). Notably, there were significant differences in the levels of TGs (TGs), phosphatidylcholine (PC), CE and other lipid components (Figure 3J and Figure S4). Moreover, the knockdown of FASN, ACC1 or ACLY partially offset the intracellular accumulation of TGs and CE, and compromised cell proliferation enhanced by CAND1 overexpression (Figure 3K,L). These observations are consistent with previous studies on CAND1 function in adipogenesis.^32^, ^33^ To analyze the involvement of CAND1 in lipid metabolism, Huh‐7 and Hep3B cells were treated with fatostatin. Fatostatin obviously reversed the effect of CAND1 on cellular proliferation which suggests that CAND1 may promote tumour cell proliferation by regulating lipid synthesis (Figure S5A,B). In addition, we repeated the cell phenotype experiments in syngeneic murine HCC cell line Hep 1–6. The results show that CAND1 promotes the proliferation, migration and lipid synthesis of Hep 1–6 (Figure S5C–G). Our findings suggest that CAND1 may promote HCC by upregulating lipid biosynthesis.

### 3.4 CAND1 functions by regulating the SCF^FBXO11^ complex to recruit hnRNPA2B1

**FIGURE 4:**
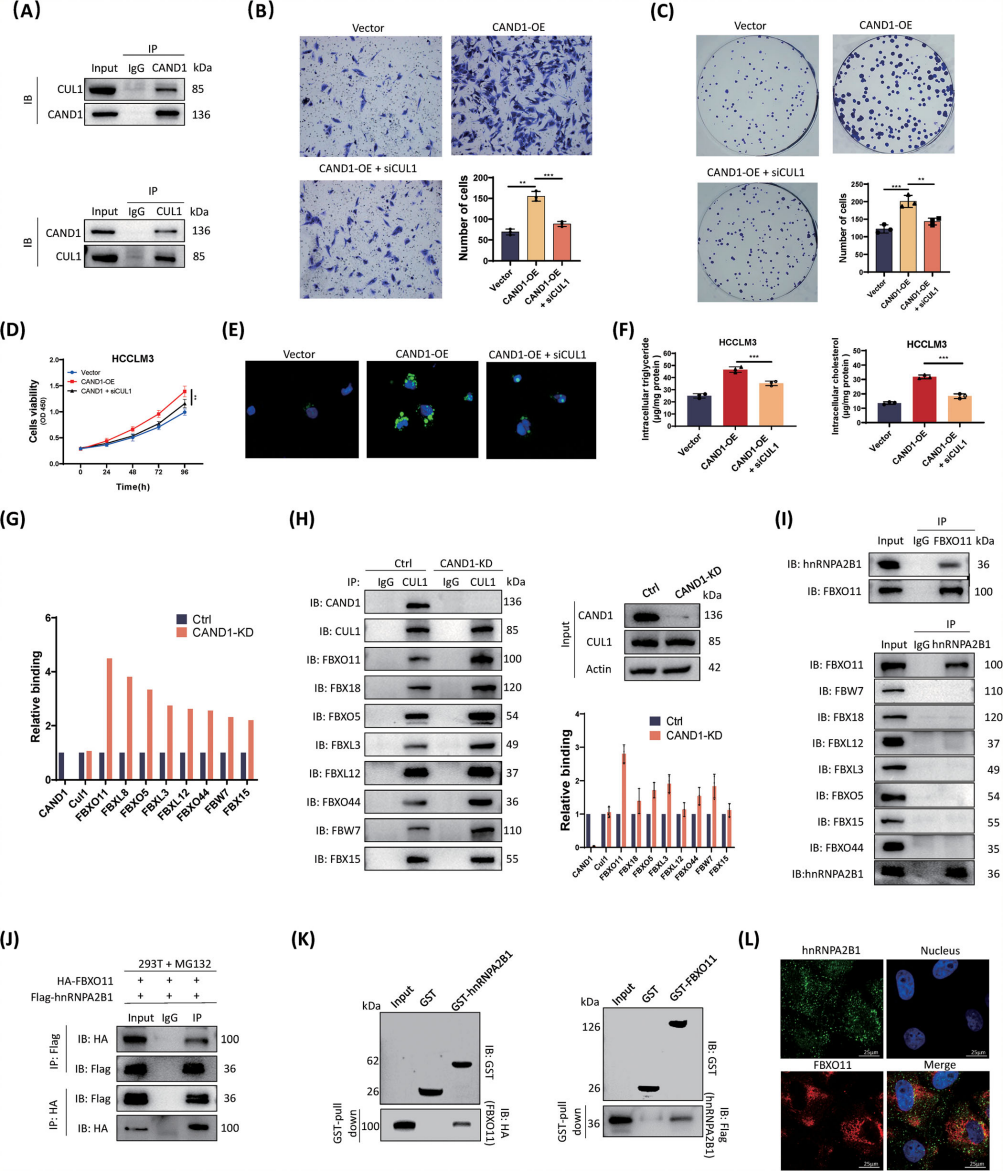
Cullin‐associated and neddylation‐dissociated 1 (CAND1) functions by regulating the SCFFBXO11 complex to recruit hnRNPA2B1. (A) A coimmunoprecipitation (co‐IP) experiment detected binding between CAND1 and CUL1. (B) CUL1 knockdown partially reversed the CAND1 overexpression‐induced promotion of cell invasion. (C) CUL1 knockdown reduced colony formation in CAND1 overexpressing HCC‐LM3 cells. (D) CCK8 assay showing that CUL1 knockdown reduces the proliferation of CAND1‐overexpressing cells. (E) BODIPY staining showed that lipid accumulation clearly decreased in the CUL1 knockdown group. (F) CUL1 knockdown partially reversed the CAND1 overexpression‐induced increase in intracellular triglycerides and cholesterol. (G) Relative quantitative mass spectrometry (MS)‐based proteomic analysis. (H) A co‐IP experiment was performed to detect binding between CUL1 and different F‐box proteins. (I) A co‐IP experiment was performed to detect binding between FBXO11 and hnRNPA2B1, and binding between hnRNPA2B1 and other F‐box proteins. (J) GST pull‐down assay. (K) A co‐IP experiment was performed to detect binding between HA‐FBXO11 and Flag‐hnRNPA2B1. (L) FBXO11 immunolabeling shows colocalization with hnRNPA2B1. All cellular experiments were run in triplicate and repeated three times. *p* < .05(*), *p* < .01(**) or *p* < .001(***).

To investigate the potential functions of the SCF complexes in HCC, we first characterized SCF complexes by performing immunoprecipitation with specific antibodies against CAND1 followed by MS (IP/MS) (Table S1). A previous study had shown that CAND1 regulated the assembly and dissociation of SCFs.^14^, ^19^ However, the mechanism by which CAND1 contributes to tumour progression and lipid metabolism through the SCF complex remains elusive. Thus, we noticed and confirmed that CUL1 is a CAND1‐interacting protein (Figure 4A). CUL1 knockdown reversed the promoting effect of CAND1 overexpression on cell proliferation, colony formation, invasion, and intracellular accumulation of lipids (Figure 4B–F and Figure S5H). These results suggest that SCF complexes are required for CAND1 functions in HCC. To map which SCF complexes were regulated by CAND1, we knocked down CAND1, performed immunoprecipitation with CUL1 antibody and measured different FBPs (Table S2). As expected, CAND1 downregulation increased the binding of multiple FBPs to CUL1 and FBXO11 showed the highest increase compared with control cells (Figure 4G,H and Figure S6A). In contrast, overexpression of CAND1 reduced CUL1 binding to FBXO11 (Figure S6B). Furthermore, CAND1 cannot decrease the CUL1 protein level (Figure S6C,D). Then, we detected the FBXO11 protein degradation rate. The results showed that the knockdown of CAND1 did not affect the degradation rate of FBXO11 protein (Figure S6E). These data suggest that CAND1 mainly regulates the SCF^FBXO11^ complex in HCC. Since FBXO11 specifically recognizes ubiquitylated substrates, we performed immunoprecipitation with FBXO11 antibody followed by MS and identified hnRNPA2B1 in the FBXO11 coprecipitate (Table S3). Among identified proteins, hnRNPA2B1 was highly expressed in HCC tissues, and its expression correlated with worse clinical prognosis (Figure S7A,B). Moreover, reports revealed that hnRNPA2B1 can regulate the expression of ACC1 and ACLY which were decreased in CAND1 knockdown cells. Thus, we chose hnRNPA2B1 as the downstream target of FBXO11.^34^ We confirmed the interaction between FBXO11 and hnRNPA2B1 in Huh‐7 cells. hnRNPA2B1 was recognized only by FBXO11, but not by other FBPs (Figure 4I). Flag‐hnRNPA2B1 and HA‐FBXO11 plasmids were cotransfected into HEK‐293T cells, and Co‐IP experiments were conducted. The results also proved the binding of FBXO11 with hnRNPA2B1 (Figure 4J). The direct interaction of FBXO11 and hnRNPA2B1 was verified by GST pull‐down assay with purified GST‐hnRNPA2B1 and HA‐FBXO11 fusion proteins expressed in E. coli (Figure 4K). Immunofluorescence staining showed the subcellular nuclear colocalization of FBXO11 (red) and hnRNPA2B1 (green). FBXO11 localized in the cytoplasm, while hnRNPA2B1 was in the nucleus and the cytoplasm (Figure 4L). Previous studies reported hnRNPA2B1 as an RNA regulator of ACLY and ACC1,^34^ which is consistent with our RNA‐Seq results and lipid accumulation phenotypes.

### 3.5 The hnRNPA2B1 mediates the regulation of CAND1 on the synthesis of lipids, proliferation and migration of tumour cells

**FIGURE 5:**
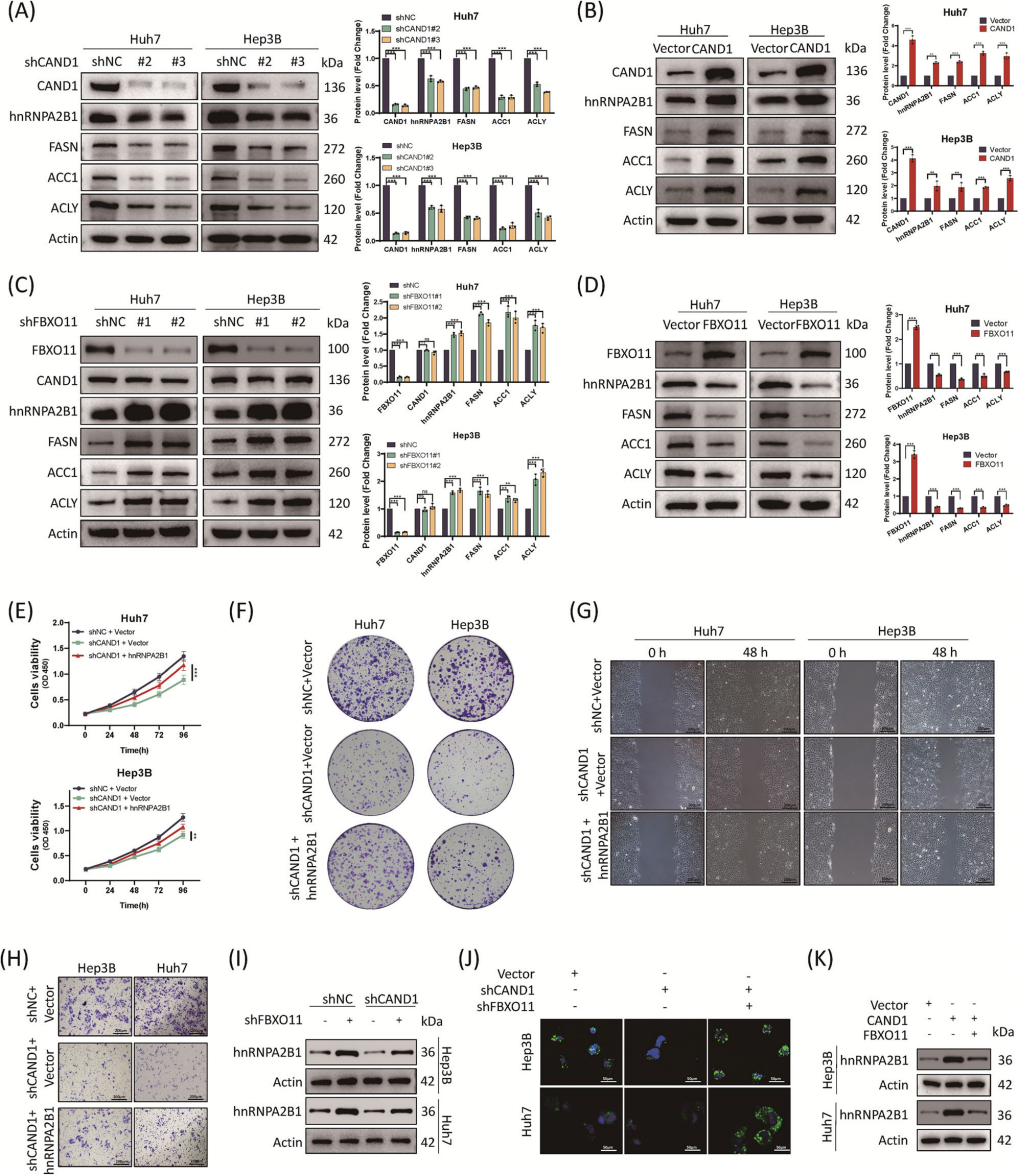
hnRNPA2B1 mediates cullin‐associated and neddylation‐dissociated 1 (CAND1) function that was antagonized by FBXO11. (A) Protein expression was assessed by a western blot of cells with CAND1 expression knocked down. (B) The expression of hnRNPA2B1, FASN, ACC1 and ACLY increased when CAND1 was overexpressed. (C) Protein expression was assessed with cells in FBXO11 expression knocked down. (D) Protein expression in cells with FBXO11 expression knocked down. (E) A Cell Counting Kit‐8 (CCK‐8) assay suggesting that hnRNPA2B1 overexpression reverses the inhibition of cell proliferation mediated by CAND1 knockdown. (F) Colony formation assays demonstrate that overexpression of hnRNPA2B1 partially reversed the suppressed proliferation induced by CAND1 knockdown. (G‐H) CAND1 expression knockdown effects on cell migration and invasion were partially reversed by hnRNPA2B1 overexpression. (I) CAND1 knockdown downregulates hnRNPA2B1 expression, which was reversed by shFBXO11. (J) CAND1 promotes lipid synthesis, which is reversed by FBXO11. (K) CAND1 overexpression upregulates hnRNPA2B1 expression, which is reversed by overexpression of FBXO11. All cellular experiments were run in triplicate and repeated three times. *p* < .05(*), *p* < .01(**) or *p* < .001(***).

The SCF^FBXO11^ complex causes degradation of the substrate. Indeed, CAND1 knockdown caused downregulation of hnRNPA2B1 and FASN, ACC, and ACLY whereas CAND1 overexpression upregulated the expression of these genes (Figure 5A,B). In contrast, we constructed FBXO11 knockdown cell lines (Figure S7C,D) and found that FBXO11 knockdown upregulated the expression of hnRNPA2B1 FASN, ACC and ACLY whereas FBXO11 overexpression repressed the expression of these genes and accumulation of lipids (Figure 5C,D and Figure S8A). More importantly, hnRNPA2B1 overexpression rescued the impairments in cell proliferation, colony formation, migration and invasion caused by CAND1 deficiency (Figure 5E–H). FBXO11 knockdown partially reversed the CAND1 knockdown‐induced downregulation of hnRNPA2B1 and accumulation of lipids (Figure 5I,J). Consistently, FBXO11 overexpression offset the upregulation of hnRNPA2B1 by CAND1 overexpression (Figure 5K). Although CAND1 can regulate hnRNAP2B1 protein expression, it cannot affect hnRNAP2B1 mRNA levels (Figure S8B–G). In addition, we performed rescue experiments in a mouse subcutaneous tumour model. Knockdown of CAND1 inhibited the growth of subcutaneous tumours in mice, while overexpression of hnRNPA2B1 partially reversed the inhibitory effect of CAND1 on tumour growth (Figure S9A).

### 3.6 CAND1 suppresses SCF^FBXO11^ complex‐mediated hnRNPA2B1 ubiquitination and degradation

**FIGURE 6:**
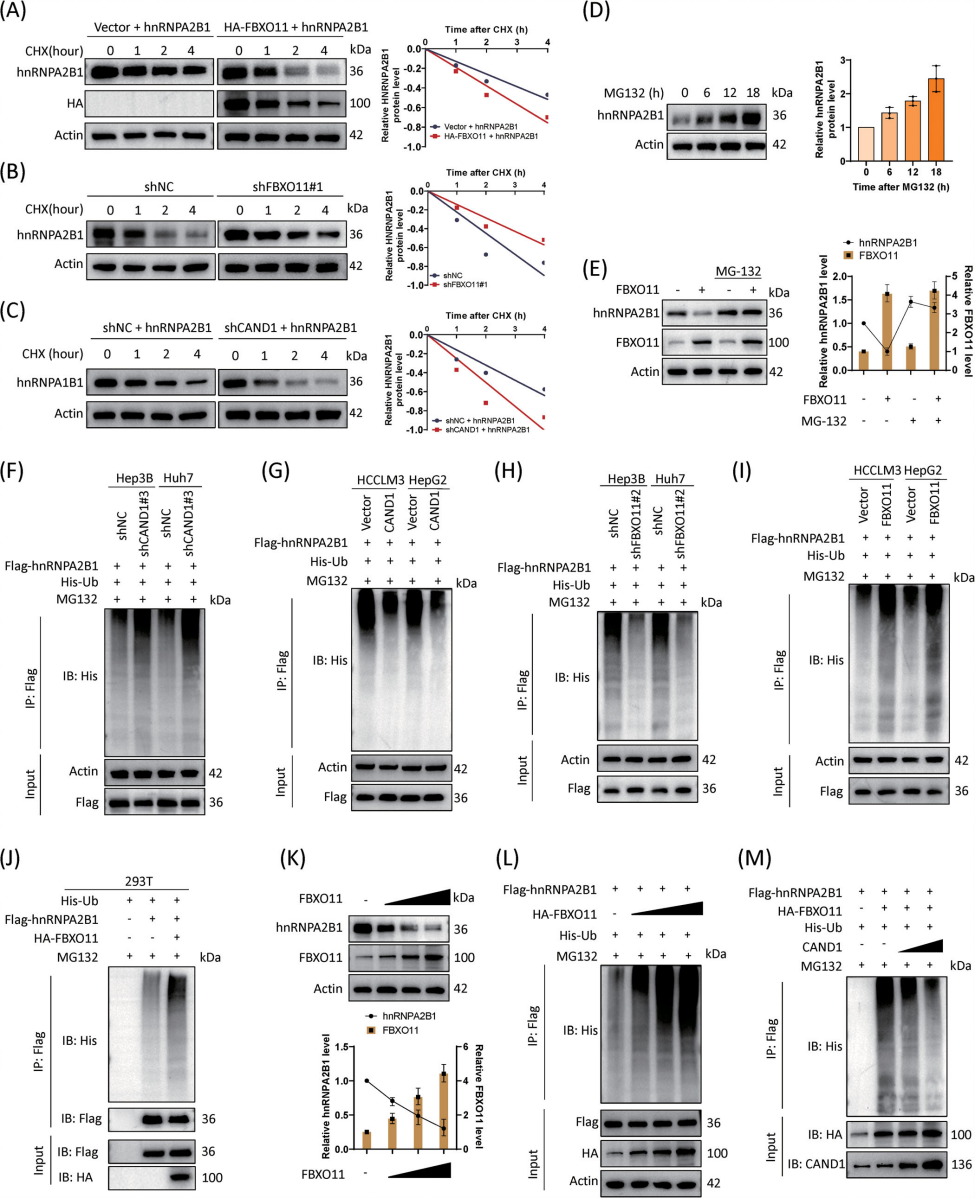
Cullin‐associated and neddylation‐dissociated 1 (CAND1) suppresses SCFFBXO11 complex‐mediated hnRNPA2B1 ubiquitination and degradation. (A) FBXO11 overexpression accelerates hnRNPA2B1 degradation. (B) FBXO11 expression knockdown decelerates hnRNPA2B1 degradation. (C) Knocking down CAND1 expression accelerates hnRNPA2B1 degradation. (D) MG132 significantly increases hnRNPA2B1 protein levels. (E) FBXO11‐induced degradation of hnRNPA2B1 is reversed by MG132 treatment. (F) The ubiquitination of hnRNPA2B1 is promoted by CAND1 expression knockdown. (G) The ubiquitination of hnRNPA2B1 is inhibited by CAND1 overexpression. (H) shRNA knockdown of FBXO11 expression levels decreased hnRNPA2B1 ubiquitination. (I) Overexpression of FBXO11 increased hnRNPA2B1 ubiquitination. (J) FBXO11 promotes hnRNPA2B1 ubiquitination in 293T cells. (K) hnRNPA2B1 protein levels are regulated by FBXO11 in a dose‐dependent manner. (L) Ubiquitination levels of hnRNPA2B1 are increased by FBXO11 in a dose‐dependent manner. (M) hnRNPA2B1 ubiquitination is regulated by CAND1 in a dose‐dependent manner. The experiments were dependently repeated three times.

FBXO11 mediates the degradation of several substrates.^35^, ^36^ We showed that FBXO11 overexpression significantly accelerated hnRNPA2B1 degradation whereas FBXO11 knockdown slowed hnRNPA2B1 degradation (Figure 6A,B). Consistently, CAND1 knockdown promoted hnRNPA2B1 degradation (Figure 6C). Moreover, treatment with the proteasome inhibitor MG132 increased hnRNPA2B1 protein levels and normalized FBXO11‐induced hnRNPA2B1 degradation (Figure 6D,E). These findings suggest that CAND1 may repress SCF^FBXO11^ complex‐induced hnRNPA2B1 degradation through ubiquitination. Indeed, hnRNPA2B1 ubiquitination was significantly increased in CAND1‐knockdown cells but significantly decreased after CAND1 overexpression (Figure 6F,G). In contrast, FBXO11 had the opposite effects (Figure 6H,I and Figure S9B). FBXO11 overexpression decreased the levels of endogenous hnRNPA2B1 protein expression and elevated the ubiquitination levels of hnRNPA2B1 in a dose‐dependent manner (Figure 6J–L). More importantly, CAND1 overexpression counteracted the effect of FBXO11 on hnRNPA2B1 ubiquitination (Figure 6 M). In summary, these results suggest that CAND1 suppresses SCF^FBXO11^ complex‐mediated hnRNPA2B1 ubiquitination and degradation.

### 3.7 FBXO11 promotes K48‐ and K27‐linked ubiquitination of hnRNPA2B1

**FIGURE 7:**
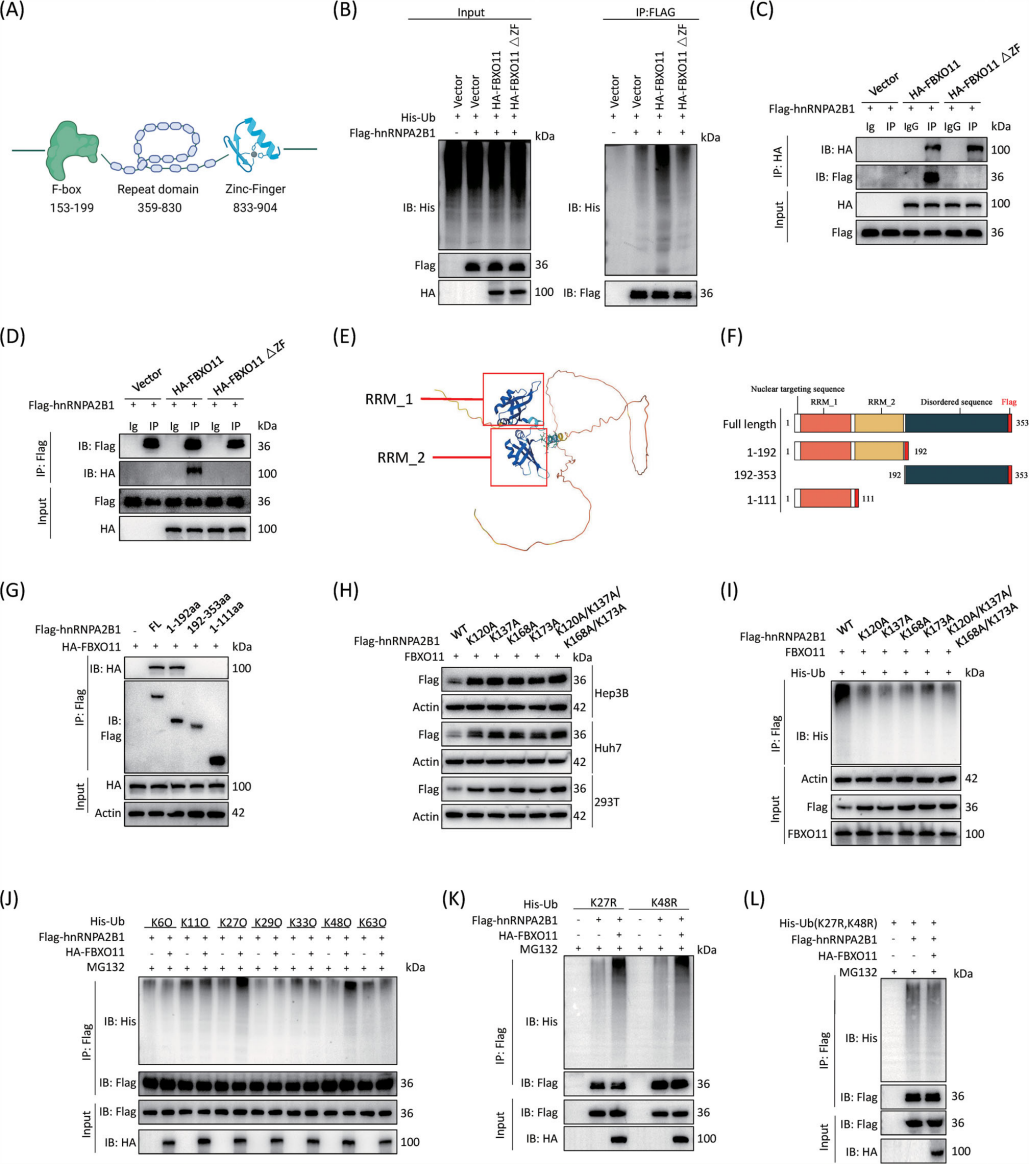
FBXO11 directly binds to and promotes K27‐ and K48‐linked ubiquitination of hnRNPA2B1. (A) Domain architectures in FBXO11 proteins. (B) FBXO11 and mutant FBXO11 were overexpressed, and ubiquitination of hnRNPA2B1 was detected. (C, D) A co‐IP experiment was performed to detect the binding of hnRNPA2B1 to FBXO11 and mutant FBXO11. (E) Domains shown in the structure diagram of hnRNPA2B1. (F) Diagrammatic representation showing hnRNPA2B1 and its truncated forms. (G) A co‐IP experiment was performed to detect the binding of FBXO11 with hnRNPA2B1 and its truncated forms. (H) The degradation rate of mutant hnRNPA2B1 is not affected by FBXO11. (I) When FBXO11 was overexpressed, ubiquitination levels of wild‐type hnRNPA2B1 and mutant hnRNPA2B1 protein levels were detected. Compared with that of wild‐type hnRNPA2B1, the ubiquitination of mutant hnRNPA2B1 was decreased. (J) FBXO11 specifically promoted the addition of K27‐ and K48‐linked ubiquitin to hnRNPA2B1. (K) K27R and K48R ubiquitin could induce the ubiquitination of hnRNPA2B1. (L) Mutant K27 and K48 ubiquitin does not increase the extent of hnRNPA2B1 ubiquitination. The experiments were dependently repeated three times.

FBXO11 contains mainly a zinc‐finger domain, which can link target proteins to a ubiquitin ligase, as well as nineteen parallel beta‐helix repeat (PbH1) motifs and an F‐box domain (Figure 7A). We generated a truncated FBXO11 without a zinc‐finger domain (833–904 amino acids) (FBXO11ΔZF). Overexpression of FBXO11 increased the ubiquitination of hnRNPA2B1, but FBXO11ΔZF did not (Figure 7B). Moreover, FBXO11ΔZF failed to interact with hnRNPA2B1 (Figure 7C,D). hnRNPA2B1 has RNA recognition motif (RRM) domains (RRM_1 containing 21–104 amino acids and RRM_2 containing 112–191 amino acids), a disordered region (193–353 amino acids) and one nuclear localization signal motif (9–15 amino acids) (Figure 7E). Among the serial truncations of hnRNPA2B1 with a Flag‐tag, only a segment of 81 amino acids in the RRM_2 region interacted with FBXO11 (Figure 7F,G). These findings suggest that the zinc finger domain of FBXO11 and the RRM_2 region of hnRNPA2B1 mediate the interaction. We then identified the domains involved in the binding of CAND1 to CUL1. We constructed truncated plasmids containing different domains of CUL1 and found that both CAND1 and FBXO11 can bind to the domain of 1–400aa of CUL1 (Figure S9C,D).

To identify the exact ubiquitination sites of hnRNPA2B1, we used online software (ubibrowser.bioit) and predicted four potential sites (Figure S9E). Mutation of four lysine residues or either side of 4 lysine residues to alanine abolished hnRNPA2B1 downregulation by FBXO11 (Figure 7H). As expected, FBXO11 failed to cause ubiquitination of these mutants (Figure 7I). Moreover, FBXO11 specifically promoted the addition of ubiquitin containing only K27 (K27O) or K48 (K48O) to hnRNPA2B1 but not other types of ubiquitin (Figure 7J). In contrast, ubiquitin with both K27R and K48R mutations could not be linked to hnRNPA2B1 by FBXO11 while K27R or K48R could be linked (Figure 7K,L). These data suggest that FBXO11 promotes hnRNPA2B1 degradation through K27‐ and K48‐linked ubiquitination.

### 3.8 AAV‐shCAND1 effectively inhibited HCC as a gene therapy

**FIGURE 8:**
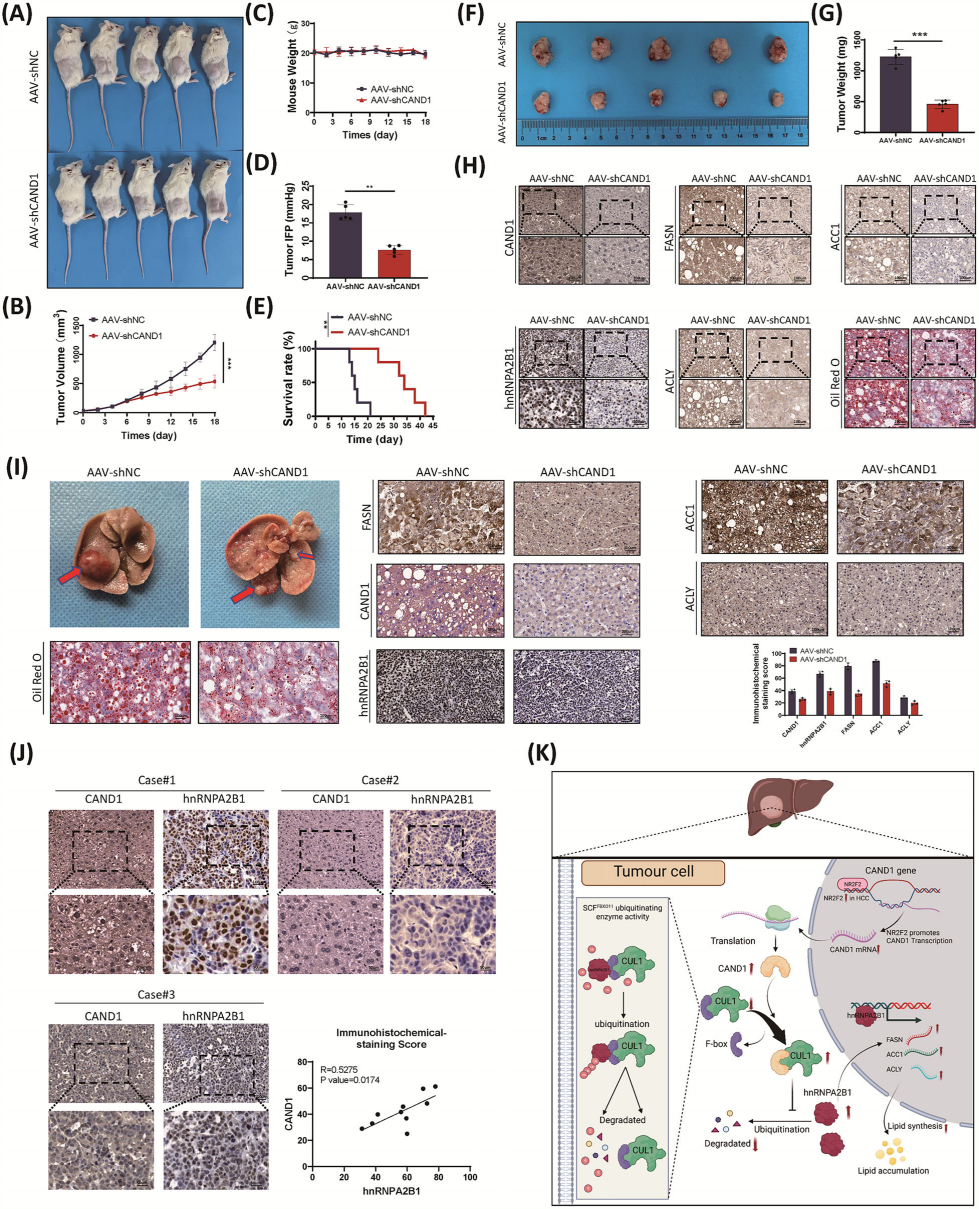
AAV‐shCAND1 effectively inhibits hepatocellular carcinoma (HCC) as a gene therapy. (A) Images of the patient‐derived xenograft (PDX) mice. (B) Plot showing tumour volume over time in the PDX mouse model. (C) Statistics of body weights of the PDX mice. (D) Interstitial fluid pressure (IFP) of tumours in PDX models. (E) Survival curve of the mice bearing PDX tumours. (F) Images of tumours in the PDX models. (G) Statistics of tumour weight of PDX mice. (H) Immunohistochemical staining of CAND1, hnRNPA2B1, FASN, ACC1 and ACLY, and oil red O staining. (I) Representative images of mouse livers with tumours induced by myr‐AKT/NRASV12 and immunohistochemical staining of fatty acid synthesis‐related proteins and oil red O staining of HCC mouse model tumour tissue. (J) IHC of tumour tissue from HCC patients. (K) Diagram of the molecular mechanisms underlying the CAND1‐SCF^FBXO11^‐hnRNPA2B1 axis. *p* < .05(*), *p* < .01(**) or *p* < .001(***).

We used a PDX mouse model to test whether targeting CAND1 was an effective strategy against HCC. Intratumoral injection of AAV‐shCAND1 potently downregulated CAND1 expression inhibited tumour growth, and decreased tumour volume without altering of mouse body weight (Figure 8A–C). Injection of AAV‐shCAND1 also decreased IFP in the centre of the tumours (Figure 8D). Moreover, the survival of nude mice was significantly improved in the group injected with AAV‐shCAND1 compared to that in the control group (Figure 8E). Furthermore, PDX tumours with intratumoral injection of AAV‐shCAND1 were smaller in size and weighed less than those in the AAV‐shNC group (Figure 8F,G). Significant decreases in CAND1, hnRNPA2B1, FASN, ACC1 and ACLY were confirmed in the AAV‐shCAND1 group (Figure 8H). To test the strategy, we also used a myr‐AKT/NRASV12‐induced mouse liver cancer model, which had abnormal lipid metabolism. The mice injected with AAV‐shCAND1 had decreased tumour sizes and reduced lipid accumulation compared to the control mice. Intratumoral injection of AAV‐shCAND1 decreased CAND1, hnRNPA2B1, FASN, ACC1, ACLY, and lipid accumulation (Figure 8I). In addition, we evaluated the expression level correlation of CAND1 and hnRNPA2B1 in clinical samples by IHC. The results showed that the protein expression of CAND1 and hnRNPA2B1 was positively correlated (R = 0.5275, Figure 8J). These results suggest that targeting CAND1 by AAV‐shCAND1 potently reduces lipogenesis and suppresses HCC in vivo.

## 4 DISCUSSION

The reprogramming of lipid metabolism is one of the characteristic features of HCC and enhanced lipogenesis has been shown to be critical for hepatocarcinogenesis.^2^, ^37^ Although several lipogenic enzymes have been shown to be upregulated in HCC, the mechanisms mediating upregulation remain to be addressed. In this study, we revealed a novel CAND1‐SCF^FBXO11^‐hnRNA2B1 axis that may enhance lipogenesis and promote HCC (Figure 8K). Our study presents new insights into the dysregulation of lipid metabolism in HCC.

CAND1‐dependent control of cullin 1‐RING ubiquitin ligases is essential for adipogenesis.^33^, ^38^ Silencing of CAND1 leads to retardation of adipogenesis. The reason for the elevated expression of CAND1 in tumours has not been previously reported. In this study, we found NR2F2 could bind the CAND1 promoter region and promote CAND1 transcription. In addition, in subsequent studies, mutation or deletion of the binding site of the CAND1 promoter region should be performed for validation. Overexpression of CAND1 increased the number of LDs in cells. Therefore, we speculated that CAND1 promotes LD formation and inhibits their degradation. However, this study has some limitations regarding fat metabolism. We did not exclude the impact of CAND1 expression on lipid uptake, fatty acid esterification or other lipid metabolic processes. More in‐depth research is needed.

Previous studies on the mechanism of CAND1 have focused mostly on the regulation of SCF ubiquitin ligase assembly by CAND1.^19^, ^39^, ^40^ However, little research has been performed to determine the mechanism by which CAND1 promotes tumour progression and lipid synthesis through the SCF complex. Through RNA sequencing analysis, we speculated that CAND1 is closely related to metabolism. However, KEGG enrichment analysis of sequencing results also showed that CAND1 may be related to the tumour necrosis factor signalling pathway, NF‐kappa B signalling pathway, and MAPK signalling pathway. Therefore, we examined related pathways and found that CAND1 can regulate P65 protein levels and caspase 8 cleavage (Figure S10A). The results show that the biological role of CAND1 is not only limited to regulating the SCFFBXO11‐hnRNPA2B1 axis but can also regulate a variety of signalling pathways in cells and play a variety of biological roles. We will explore other biological functions and molecular mechanisms of CAND1 in depth in subsequent studies. Furthermore, the present evidence clearly demonstrates that SCF^FBXO11^ is the main factor affected by CAND1 in HCC but does not exclude other SCF complexes that may also play roles in HCC.

Our study demonstrates that the RNA‐binding protein hnRNPA2B1 is the substrate for SCF^FBXO11^ to perform its function. hnRNPA2B1 engages in many important cellular activities through transcription regulation, splicing processing, RNA transport, translation and other processes.^41^ hnRNPA2B1 can promote the progression of several tumour types by a variety of mechanisms.^30^, ^42^, ^43^ Moreover, hnRNPA2B1 contributed to tumour progression by upregulating enzymes involved in lipid metabolism, such as ACLY and acetyl‐CoA carboxylase 1 (ACC1).^34^ Consistent with these findings, our sequencing results showed that ACLY and ACC1 were downregulated in CAND1 knockdown cells. Moreover, we verified that hnRNPA2B1 mediated CAND1 function in tumour progression and lipid metabolism. Further RIP‐Seq may provide direct evidence that hnRNPA2B1 targets lipid biosynthesis genes. In addition, CAND2 is thought to have similar roles to CAND1.^44^, ^45^ One study reported an association of CAND2 with the prognosis of HCC.^46^ However, we found that knocking down CAND2 in Huh‐7 cells had no effect on the levels of hnRNPA2B1 mRNA and protein (Figure S10B–D).

Our study provides a novel strategy against HCC. We demonstrate that the CAND1‐SCF^FBXO11^‐hnRNPA2B1 axis promotes lipid accumulation and contributes to the progression of HCC. In liver cancer PDX mouse models and primary HCC mouse models, CAND1 knockdown significantly inhibited lipid accumulation and tumour growth in vivo. However, this research still has many deficiencies. First, glucose metabolism is an important part of metabolism. We focused on lipid metabolism but did not conduct in‐depth research on glucose metabolism. Secondly, we determined the biological functions of CAND1 through mRNA sequencing and lipid metabolomics. If proteomics and single‐cell sequencing are added, the functions of CAND1 will be better explored. Finally, in addition to using AAVs to knock down CAND1, a small molecule compound library can also be used to screen small molecule inhibitors targeting CAND1. This is also worthy of further study in the future.

In summary, our study unveils a novel CAND1‐SCF^FBXO11^‐hnRNPA2B1 axis that enhances lipogenesis and promotes HCC. Findings obtained from AAV‐mediated gene targeting experiments were performed and suggest that AAV‐shCAND1 is highly effective against HCC in preclinical models. The regulatory axis may provide potential targets for developing new strategies for HCC therapy.

## CONFLICT OF INTEREST STATEMENT

The authors declare no conflict of interest.

## FUNDING INFORMATION

Our work was supported by the Cancer Research and Translational Platform Project of Zhongnan Hospital of Wuhan University (ZLYNXM202004), a grant from the Key Research and Development Program of Hubei Province (2021BCA114), the Research Fund of the Health Commission of Hubei Province (WJ2021M255), and Zhongnan Hospital of Wuhan University Science, Technology and Innovation Seed Fund (CXPY2023053).

## Supporting information

Supporting Information

Supporting Information

Supporting Information

Supporting Information

Supporting Information

Supporting Information

Supporting Information

Supporting Information

Supporting Information

Supporting Information

Supporting Information

Supporting Information

Supporting Information

## Data Availability

The authors confirm that the data supporting the findings of this study are available within the article. The original data presented in the study are included in the article/supplementary material and https://dataview.ncbi.nlm.nih.gov/object/PRJNA877007?reviewer = rr0tg5ql8abigc7ml6bscks4hc. Further inquiries can be directed to the corresponding author.
