# Erratum for the article “Cullin‐associated and neddylation‐dissociated 1 regulate reprogramming of lipid metabolism through SKP1‐Cullin‐1‐F‐box^FBXO11^ ‐mediated heterogeneous nuclear ribonucleoprotein A2/B1 ubiquitination and promote hepatocellular carcinoma” by Zhang H et al.

**DOI:** 10.1002/ctm2.1805

**Published:** 2024-08-07

The authors acknowledge an error in the presentation of Oil Red staining images of liver cancer tissue in the AAV‐shCAND1 group within the mouse primary liver cancer model in Figure. 8I. Figure 8I mistakenly displayed images from the AAV‐shCAND1 group of the PDX model, rather than the intended primary liver cancer model.

**FIGURE 8:**
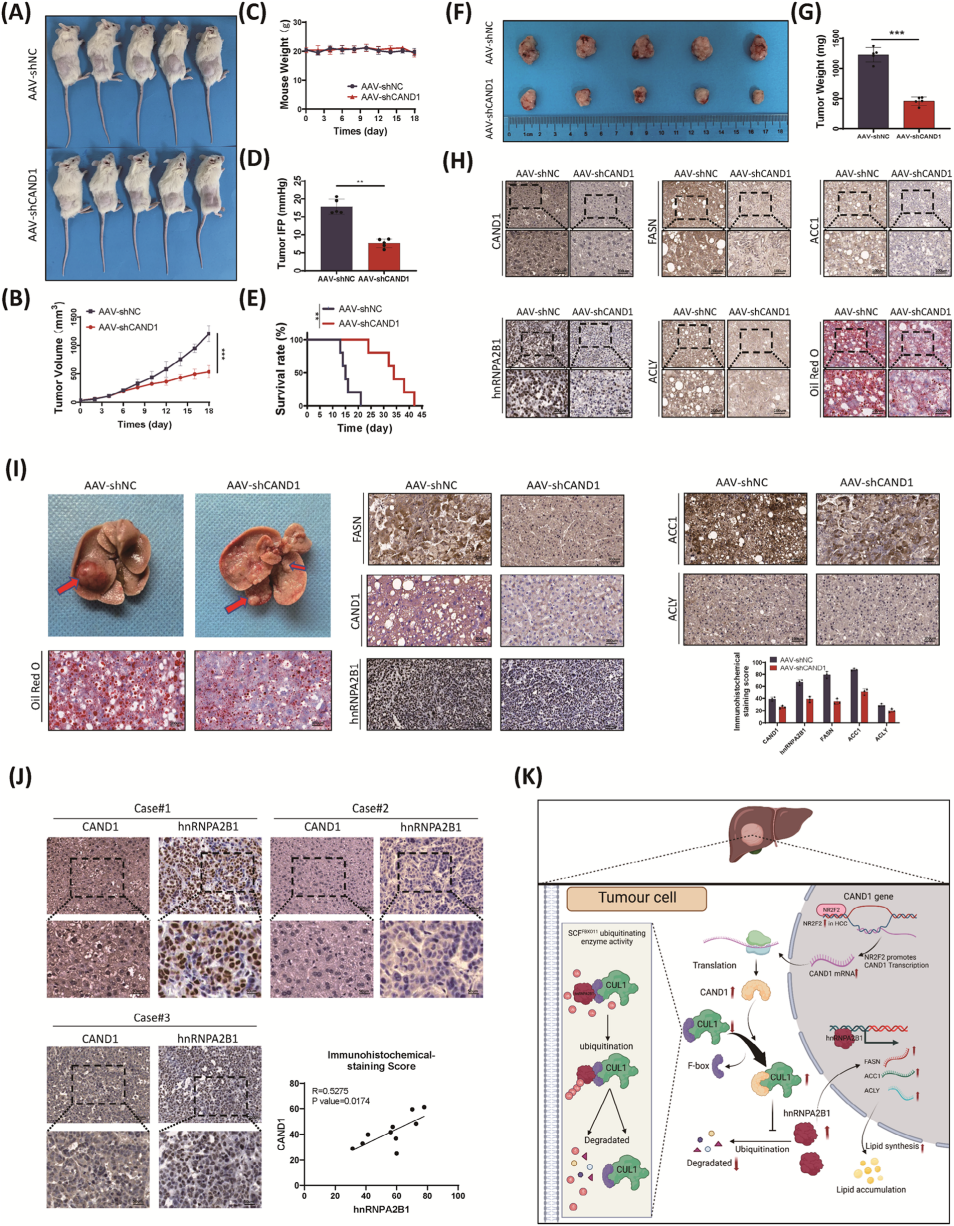


**Original Version**:

**Description**:

To test the strategy, we also used a myr‐AKT/NRASV12‐induced mouse liver cancer model, which had abnormal lipid metabolism. The mice injected with AAV‐shCAND1 had decreased tumor sizes and reduced lipid accumulation compared to the control mice. Intratumoral injection of AAV‐shCAND1 decreased CAND1, hnRNPA2B1, FASN, ACC1, ACLY, and lipid accumulation (Figure 8I).

**Picture of the original version** Figure 8 **with red block**:

We replaced the image of oil red staining of mouse liver in the AAV‐shCAND1 group in Figure 8I with a new image.

We sincerely apologize for this oversight. The corrected Figure 8, presented below, now accurately depicts the Oil Red staining results from the primary liver cancer model.

**Corrected picture**: Figure 8

In addition, the description of Figure 8 in the results section of the original version does not need to be changed.

The authors apologize for these errors and for any inconvenience caused and appreciate your understanding and support. The corrections have no impact on the experimental outcome or conclusions

