# Supplementary figures and images for: Cullin‐associated and neddylation‐dissociated 1 regulate reprogramming of lipid metabolism through SKP1‐Cullin‐1‐F‐boxFBXO11‐mediated heterogeneous nuclear ribonucleoprotein A2/B1 ubiquitination and promote hepatocellular carcinoma
Source: Clin Transl Med. 2023 Oct 14;13(10):e1443. doi: 10.1002/ctm2.1443 (PMC10576442; doi:10.1002/ctm2.1443)

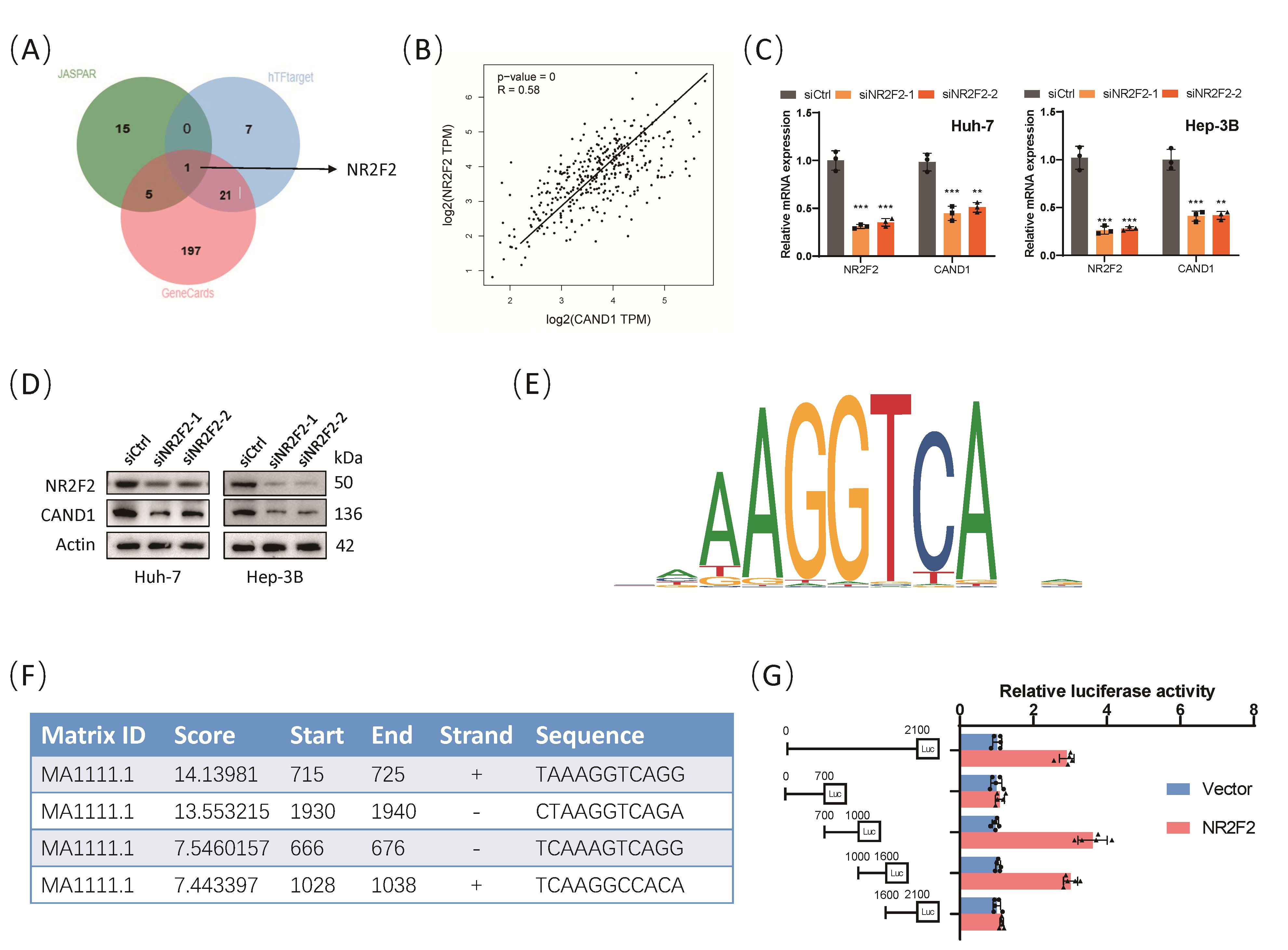

Supplement: Supplementary file 1 — Supporting Information [file CTM2-13-e1443-s006.tif]

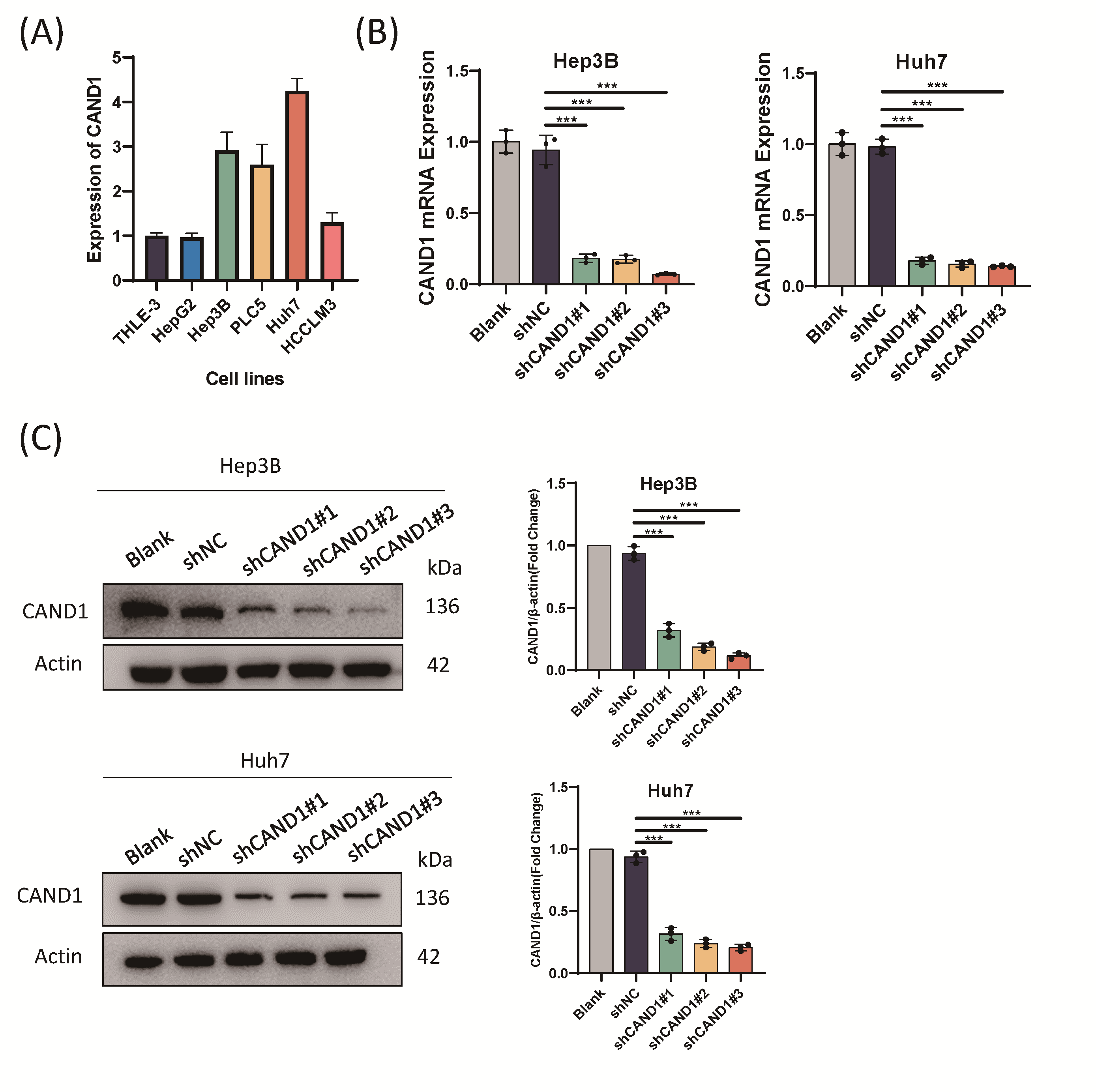

Supplement: Supplementary file 2 — Supporting Information [file CTM2-13-e1443-s005.tif]

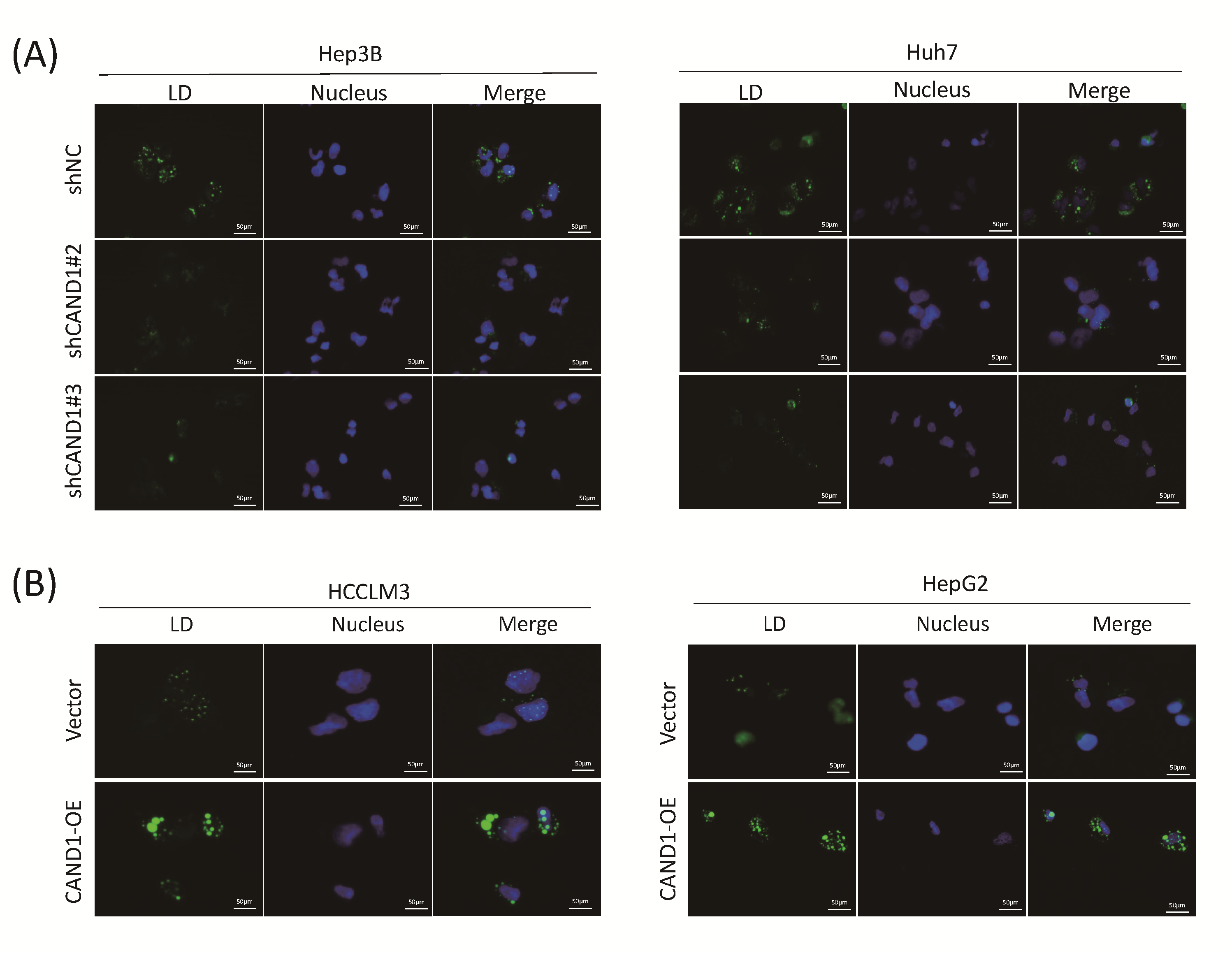

Supplement: Supplementary file 3 — Supporting Information [file CTM2-13-e1443-s001.tif]

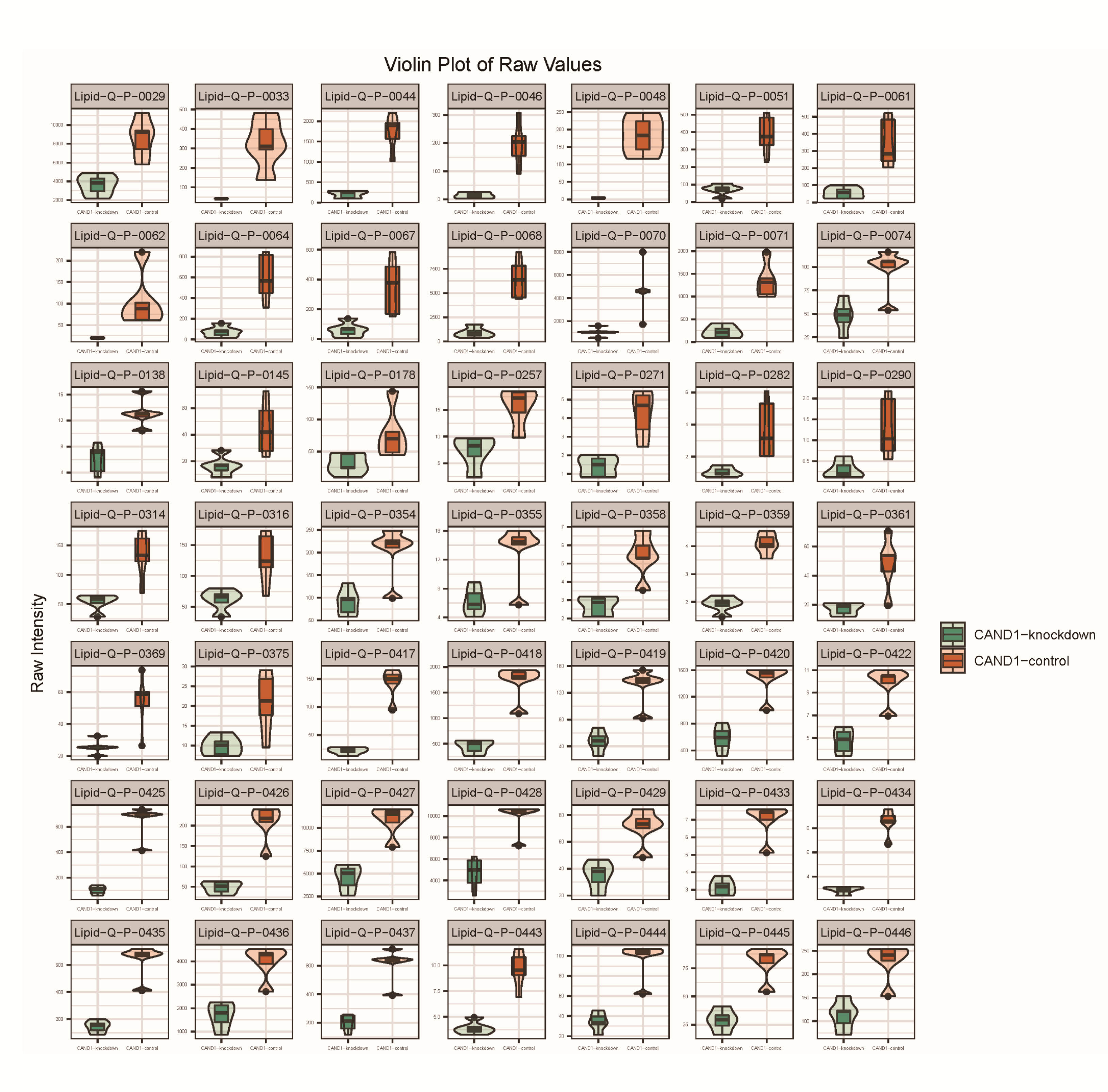

Supplement: Supplementary file 4 — Supporting Information [file CTM2-13-e1443-s003.tif]

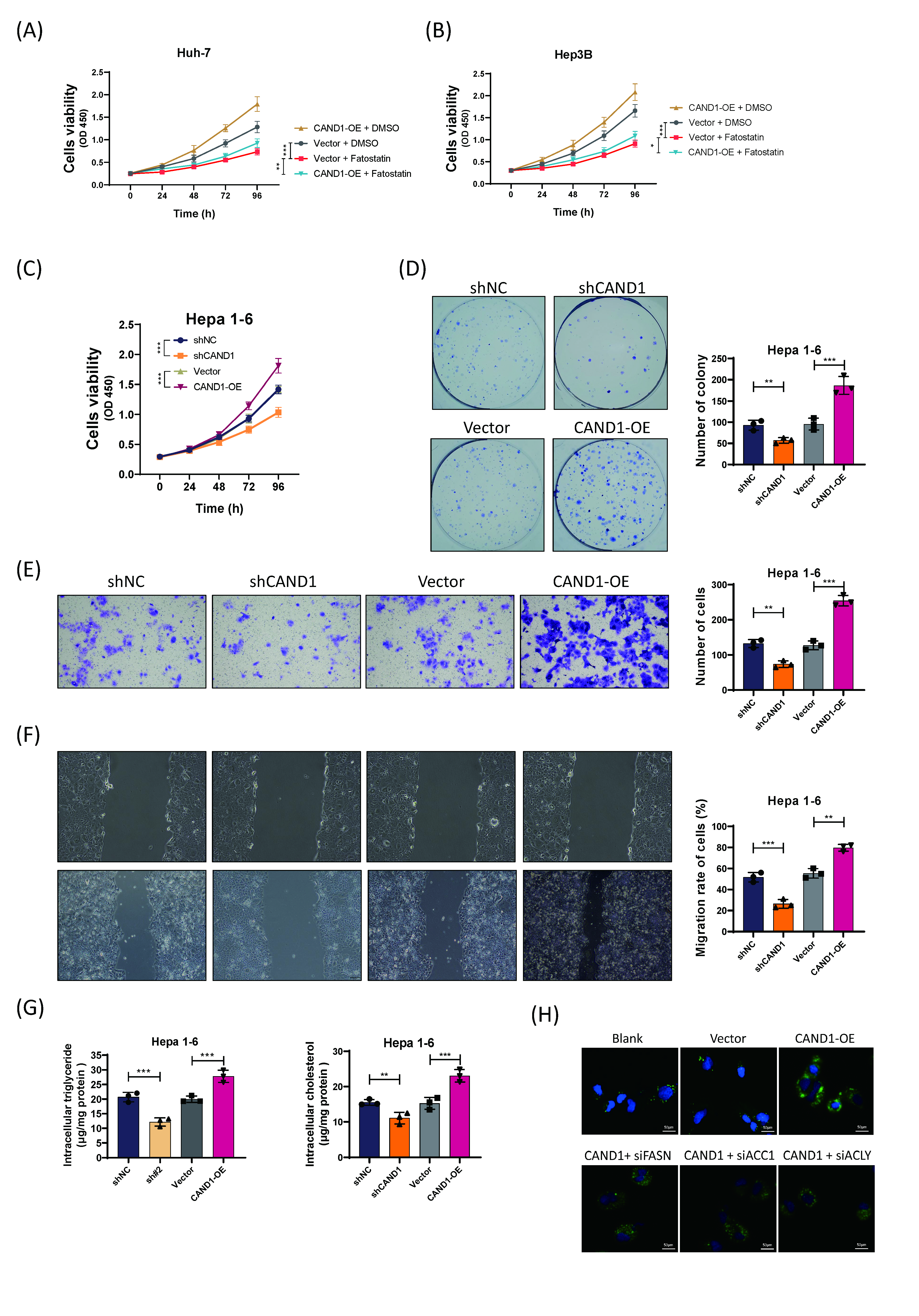

Supplement: Supplementary file 5 — Supporting Information [file CTM2-13-e1443-s008.tif]

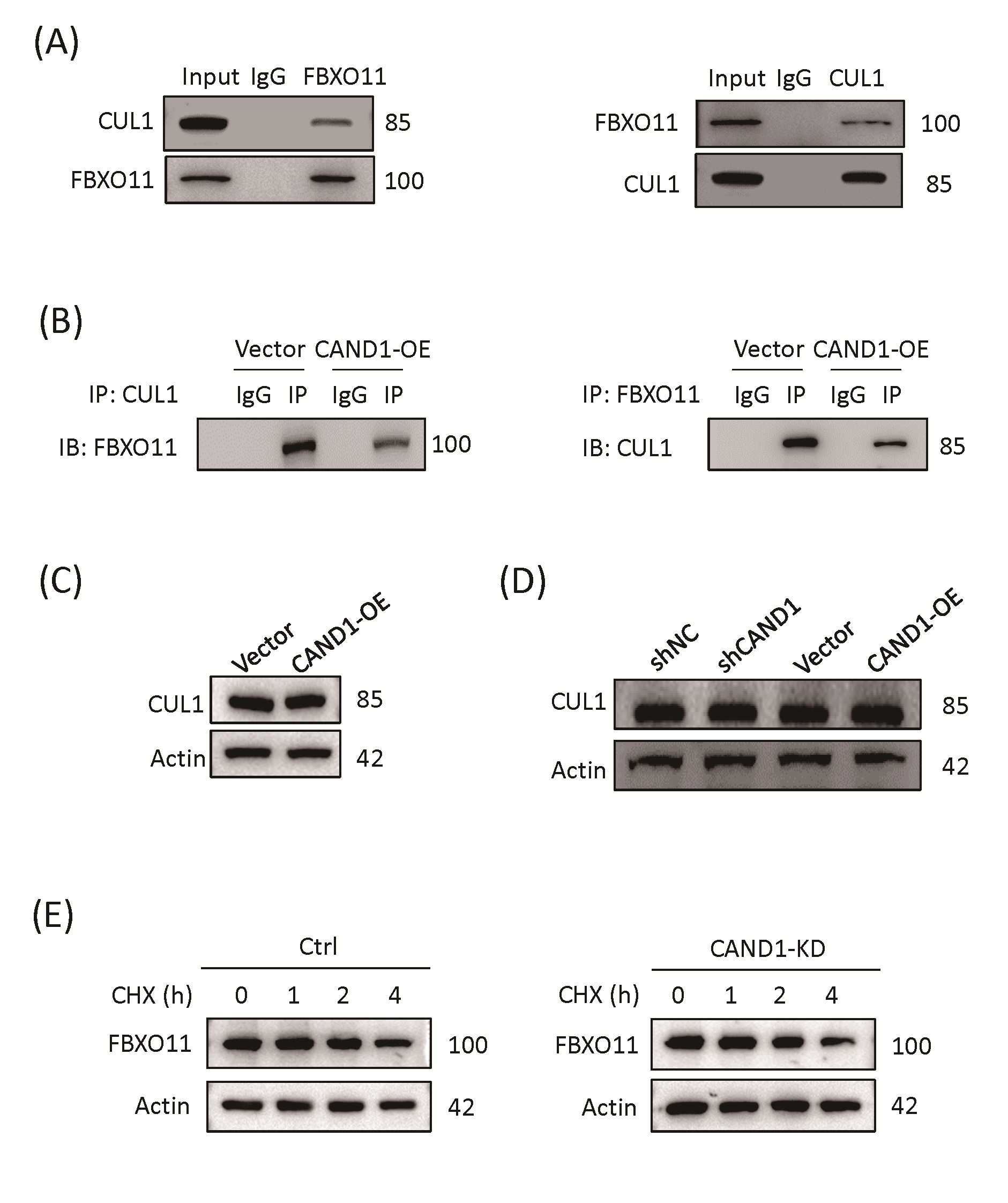

Supplement: Supplementary file 6 — Supporting Information [file CTM2-13-e1443-s009.tif]

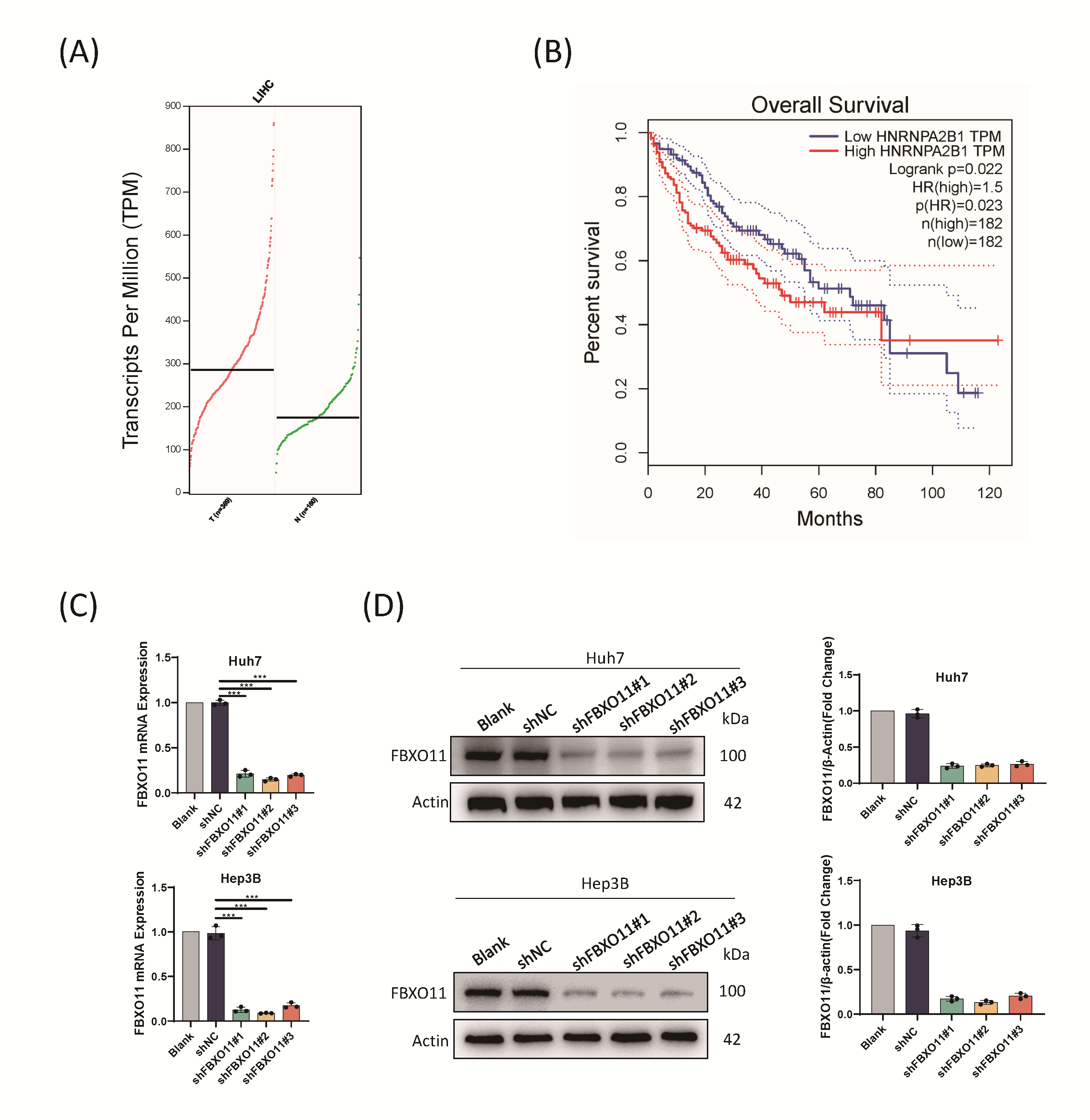

Supplement: Supplementary file 7 — Supporting Information [file CTM2-13-e1443-s002.tif]

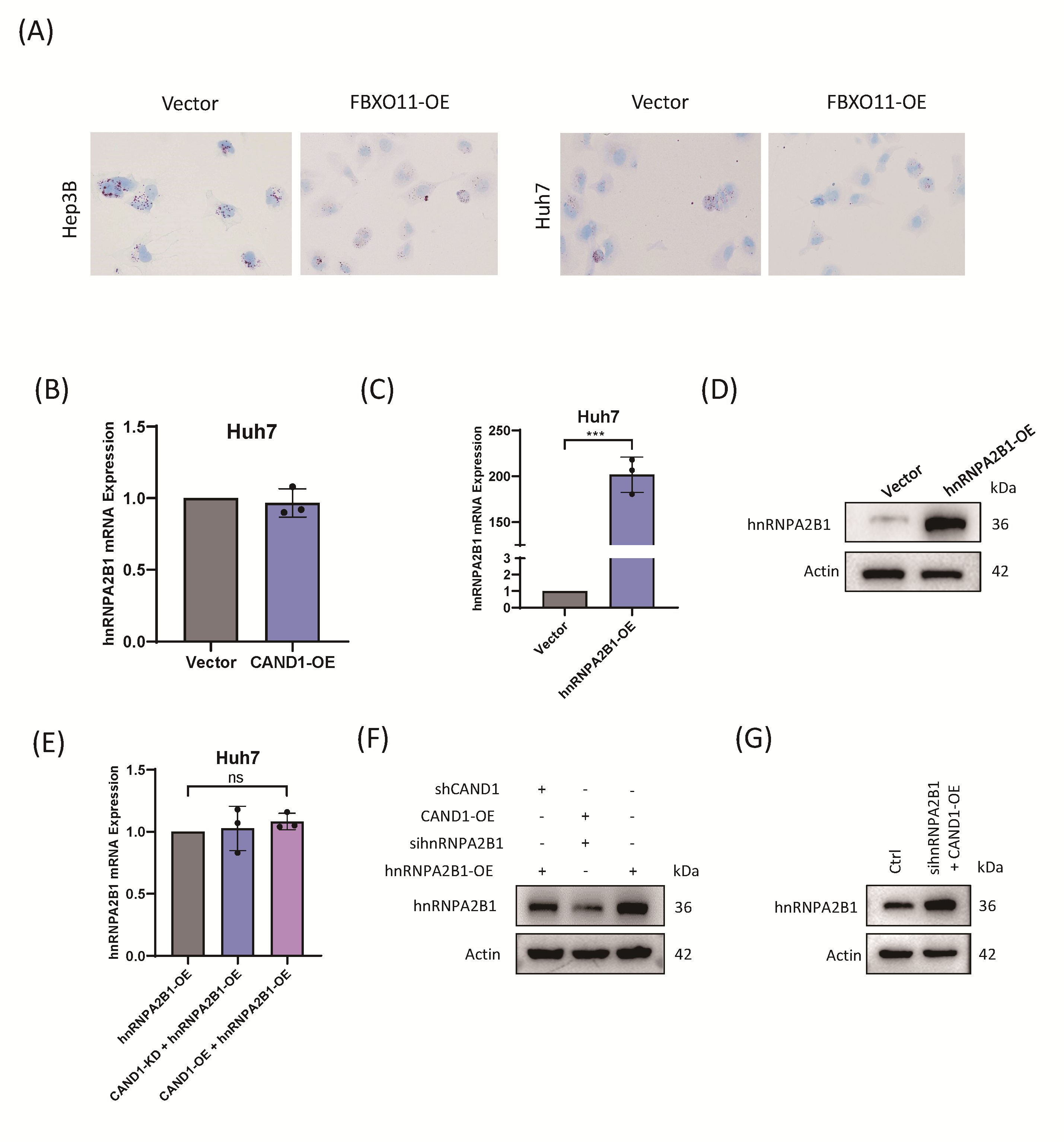

Supplement: Supplementary file 8 — Supporting Information [file CTM2-13-e1443-s012.tif]

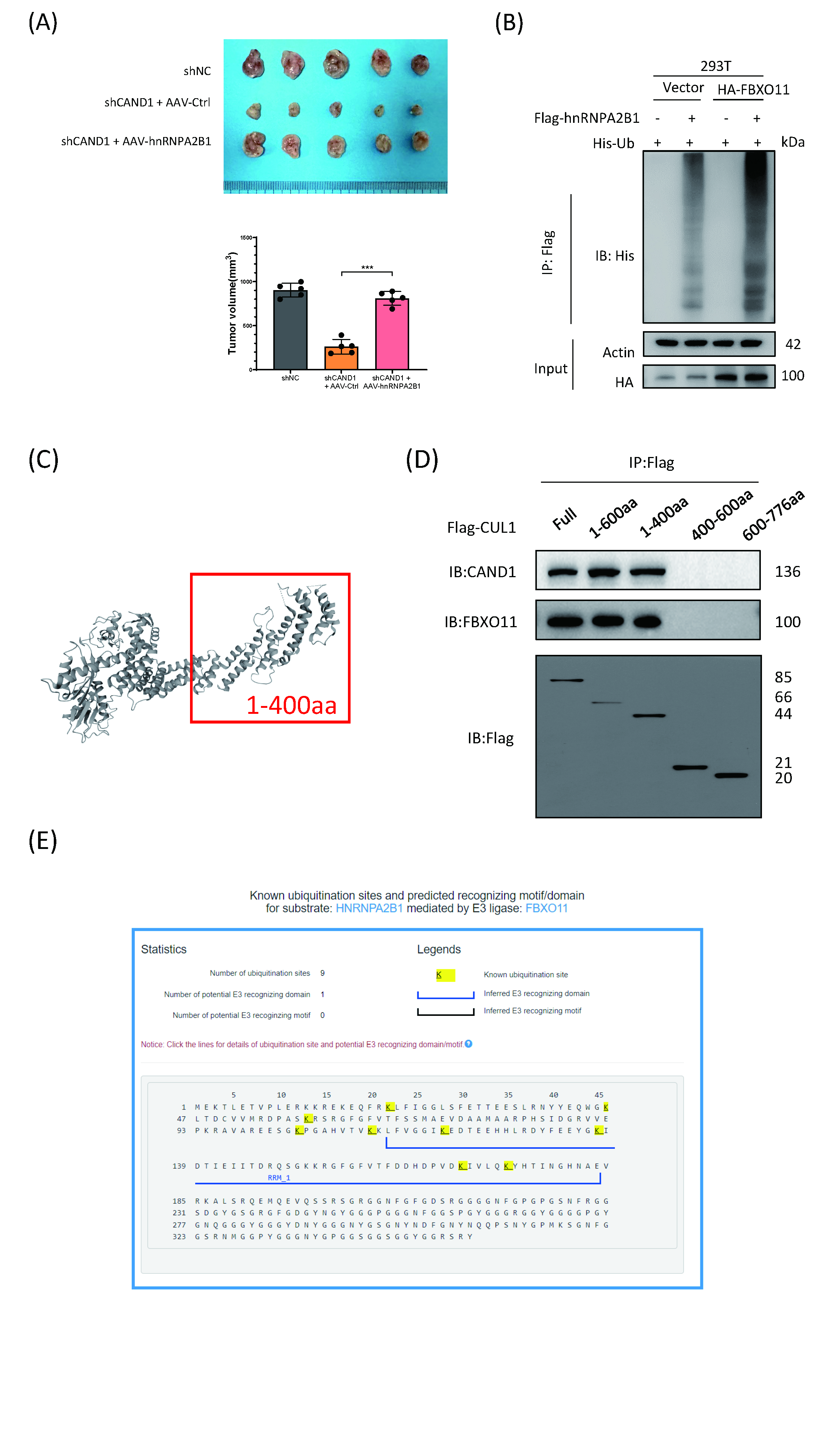

Supplement: Supplementary file 9 — Supporting Information [file CTM2-13-e1443-s007.tif]

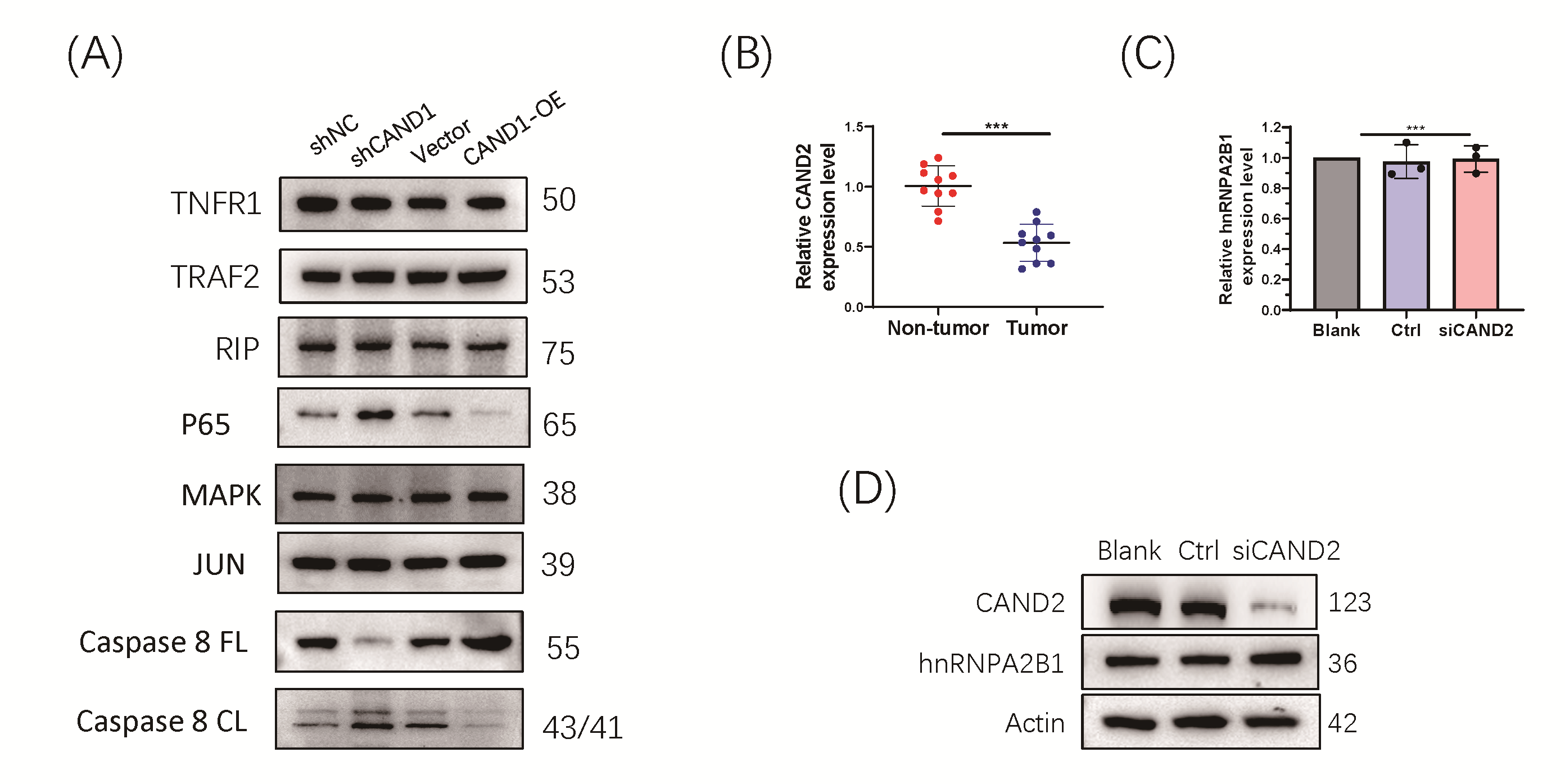

Supplement: Supplementary file 10 — Supporting Information [file CTM2-13-e1443-s013.tif]
