# Supplementary material for: Cullin‐associated and neddylation‐dissociated 1 regulate reprogramming of lipid metabolism through SKP1‐Cullin‐1‐F‐boxFBXO11‐mediated heterogeneous nuclear ribonucleoprotein A2/B1 ubiquitination and promote hepatocellular carcinoma
Source: Clin Transl Med. 2023 Oct 14;13(10):e1443. doi: 10.1002/ctm2.1443 (PMC10576442; doi:10.1002/ctm2.1443)
Supplement: Supplementary file 11 — Supporting Information [file CTM2-13-e1443-s011.pdf]

Supplementary Table. S1
Results of MS (IP/MS) following IP of CAND1 and identified CUL1 as a potential interacting protein.

Accession	Gene	Description	Mw(kDa)	CAND1	IgG	log2(CAND1/IgG	Diff Sig
A0A075B7G3	ZNF595	Zinc finger protein 595 OS=Homo sapiens OX=9606 GN=ZNF595 PE=1 SV=2	53.305	18.6852	25.3231	-6.6379	--
C9JNW0	SH3BP5	SH3 domain-binding protein 5 (Fragment) OS=Homo sapiens OX=9606 GN=S	5.9559	19.7201	25.8387	-6.1186	--
P28074	PSMB5	Proteasome subunit beta type-5 OS=Homo sapiens OX=9606 GN=PSMB5 PE	28.48	25.2618	19.2973	5.9645	++
O14818	PSMA7	Proteasome subunit alpha type-7 OS=Homo sapiens OX=9606 GN=PSMA7 P	27.887	25.2385	19.4344	5.8041	++
P54826	GAS1	Growth arrest-specific protein 1 OS=Homo sapiens OX=9606 GN=GAS1 PE=	35.693	20.1536	25.8667	-5.7131	--
Q13616	CUL1	Cullin-1 OS=Homo sapiens OX=9606 GN=CUL1 PE=1 SV=3	89.679	26.1607	20.5562	5.6045	++
B4DY09	ILF2	Interleukin enhancer-binding factor 2 OS=Homo sapiens OX=9606 GN=ILF2 F	38.91	23.4517	17.8932	5.5585	++
P60900	PSMA6	Proteasome subunit alpha type-6 OS=Homo sapiens OX=9606 GN=PSMA6 P	27.399	24.4474	18.897	5.5504	++
A0A0A0MS07	IGHG1	Immunoglobulin heavy constant gamma 1 (Fragment) OS=Homo sapiens OX=	31.983	25.3658	30.7036	-5.3378	--
Q9Y265	RUVBL1	RuvB-like 1 OS=Homo sapiens OX=9606 GN=RUVBL1 PE=1 SV=1	50.227	24.4877	19.3111	5.1766	++
E9PLY0	MACF1	Microtubule-actin cross-linking factor 1, isoforms 1/2/3/5 OS=Homo sapiens O	119.93	19.8316	24.9737	-5.1421	--
Q01469	FABP5	Fatty acid-binding protein 5 OS=Homo sapiens OX=9606 GN=FABP5 PE=1 S	15.164	23.3076	18.2281	5.0795	++
P28070	PSMB4	Proteasome subunit beta type-4 OS=Homo sapiens OX=9606 GN=PSMB4 PE	29.204	24.423	19.4487	4.9743	++
P49721	PSMB2	Proteasome subunit beta type-2 OS=Homo sapiens OX=9606 GN=PSMB2 PE	22.836	24.2038	19.5364	4.6674	+
P28072	PSMB6	Proteasome subunit beta type-6 OS=Homo sapiens OX=9606 GN=PSMB6 PE	25.357	23.4925	18.8775	4.615	+
Q99436	PSMB7	Proteasome subunit beta type-7 OS=Homo sapiens OX=9606 GN=PSMB7 PE	29.965	22.6231	18.0277	4.5954	+
Q14DU5	ROCK2	Non-specific serine/threonine protein kinase OS=Homo sapiens OX=9606 GN	82.148	24.0922	19.7561	4.3361	+
A0A075B6I1	IGLV4-60	Immunoglobulin lambda variable 4-60 OS=Homo sapiens OX=9606 GN=IGLV	12.987	22.4759	26.5655	-4.0896	--
P28066	PSMA5	Proteasome subunit alpha type-5 OS=Homo sapiens OX=9606 GN=PSMA5 P	26.411	25.672	21.6957	3.9763	+
A0A024RA52	PSMA2	Proteasome subunit alpha type OS=Homo sapiens OX=9606 GN=PSMA2 PE=	25.898	24.9541	21.061	3.8931	+
P52597	HNRNPF	Heterogeneous nuclear ribonucleoprotein F OS=Homo sapiens OX=9606 GN=	45.671	23.2724	19.4603	3.8121	+
H0YM91	TBXT	T-box transcription factor T (Fragment) OS=Homo sapiens OX=9606 GN=TBX	27.409	20.0776	23.8325	-3.7549	--
P25786	PSMA1	Proteasome subunit alpha type-1 OS=Homo sapiens OX=9606 GN=PSMA1 P	29.555	22.9222	19.2065	3.7157	+
P25788	PSMA3	Proteasome subunit alpha type-3 OS=Homo sapiens OX=9606 GN=PSMA3 P	28.433	25.6915	22.2705	3.421	+
B4DNK4	PKM	Pyruvate kinase OS=Homo sapiens OX=9606 GN=PKM PE=1 SV=1	49.897	20.9553	17.5407	3.4146	+
Q8TDY2	RB1CC1	RB1-inducible coiled-coil protein 1 OS=Homo sapiens OX=9606 GN=RB1CC1	183.09	20.3535	23.7601	-3.4066	-
E5RI98	NPM1	Nucleophosmin (Fragment) OS=Homo sapiens OX=9606 GN=NPM1 PE=1 S\	11.865	22.1506	18.9122	3.2384	+
A0A087WXQ8	PSMB3	Proteasome chain 13 OS=Homo sapiens OX=9606 GN=PSMB3 PE=1 SV=1	12.082	25.3131	22.1852	3.1279	+
P02008	HBZ	Hemoglobin subunit zeta OS=Homo sapiens OX=9606 GN=HBZ PE=1 SV=2	15.637	23.3604	20.2875	3.0729	+
B4E099	DBF4B	Protein DBF4 homolog B OS=Homo sapiens OX=9606 GN=DBF4B PE=1 SV=	16.961	25.5969	22.5411	3.0558	+
F8W079	ATP5F1B	ATP synthase subunit beta, mitochondrial (Fragment) OS=Homo sapiens OX=	30.17	22.0355	19.117	2.9185	+
Q96A65	EXOC4	Exocyst complex component 4 OS=Homo sapiens OX=9606 GN=EXOC4 PE=	110.5	19.6675	22.438	-2.7705	-
P61978	HNRNPK	Heterogeneous nuclear ribonucleoprotein K OS=Homo sapiens OX=9606 GN=	50.976	22.0812	19.383	2.6982	+
K7ELW5	PTBP1	Polypyrimidine tract-binding protein 1 (Fragment) OS=Homo sapiens OX=960	11.209	23.0836	20.7444	2.3392	+
E9PLD0	RAB1B	Ras-related protein Rab-1B OS=Homo sapiens OX=9606 GN=RAB1B PE=1 S	18.483	22.1411	19.8481	2.293	+
Q8WUM4	PDCD6IP	Programmed cell death 6-interacting protein OS=Homo sapiens OX=9606 GN	96.022	21.0876	18.8044	2.2832	+
F6UXX1	SYNCRIP	Heterogeneous nuclear ribonucleoprotein Q (Fragment) OS=Homo sapiens O	20.256	23.4736	21.4908	1.9828	+
P35579	MYH9	Myosin-9 OS=Homo sapiens OX=9606 GN=MYH9 PE=1 SV=4	226.53	19.8027	21.7653	-1.9626	-
O75342	ALOX12B	Arachidonate 12-lipoxygenase, 12R-type OS=Homo sapiens OX=9606 GN=AI	80.355	20.2801	22.1769	-1.8968	-
F8VV32	LYZ	1,4-beta-N-acetylmuramidase C OS=Homo sapiens OX=9606 GN=LYZ PE=1	11.488	22.4341	20.6546	1.7795	+
Q6ZVX7	NCCRP1	F-box only protein 50 OS=Homo sapiens OX=9606 GN=NCCRP1 PE=1 SV=1	30.847	20.5834	22.3425	-1.7591	-
P13010	XRCC5	X-ray repair cross-complementing protein 5 OS=Homo sapiens OX=9606 GN=	82.704	21.92	20.2075	1.7125	+
Q9Y490	TLN1	Talin-1 OS=Homo sapiens OX=9606 GN=TLN1 PE=1 SV=3	269.76	21.976	20.2805	1.6955	+
H0YAS6	PABPC1	Polyadenylate-binding protein 1 (Fragment) OS=Homo sapiens OX=9606 GN=	17.843	25.0657	23.4354	1.6303	+
A0A2R8Y6I8	ENO1	Alpha-enolase (Fragment) OS=Homo sapiens OX=9606 GN=ENO1 PE=1 SV=	3.5622	22.9584	21.4776	1.4808	+
P47929	LGALS7	Galectin-7 OS=Homo sapiens OX=9606 GN=LGALS7 PE=1 SV=2	15.075	21.767	23.1931	-1.4261	-
P06702	S100A9	Protein S100-A9 OS=Homo sapiens OX=9606 GN=S100A9 PE=1 SV=1	13.242	25.7	27.1109	-1.4109	-
O43390	HNRNPR	Heterogeneous nuclear ribonucleoprotein R OS=Homo sapiens OX=9606 GN=	70.942	22.612	21.2439	1.3681	+
P05109	S100A8	Protein S100-A8 OS=Homo sapiens OX=9606 GN=S100A8 PE=1 SV=1	10.834	24.7071	26.0444	-1.3373	-
P11021	HSPA5	Endoplasmic reticulum chaperone BiP OS=Homo sapiens OX=9606 GN=HSP	72.332	24.1788	22.9137	1.2651	+
C9J0S9	HSPD1	60 kDa heat shock protein, mitochondrial (Fragment) OS=Homo sapiens OX=	6.3646	22.3436	21.1046	1.239	+
P11279	LAMP1	Lysosome-associated membrane glycoprotein 1 OS=Homo sapiens OX=9606	44.882	20.292	21.5078	-1.2158	-
Q96GW1	HSP90B1	Endoplasmin OS=Homo sapiens OX=9606 GN=HSP90B1 PE=1 SV=1	35.449	23.7984	22.5991	1.1993	+
P31151	S100A7	Protein S100-A7 OS=Homo sapiens OX=9606 GN=S100A7 PE=1 SV=4	11.471	24.0681	25.2668	-1.1987	-
P11142	HSPA8	Heat shock cognate 71 kDa protein OS=Homo sapiens OX=9606 GN=HSPA8	70.897	25.4395	24.3823	1.0572	+
A0A2R8Y5P9	SHROOM3	Protein Shroom3 OS=Homo sapiens OX=9606 GN=SHROOM3 PE=1 SV=1	208.05	29.2542	30.2666	-1.0124	-
Q14847	LASP1	LIM and SH3 domain protein 1 OS=Homo sapiens OX=9606 GN=LASP1 PE=	29.717	27.3939	26.4273	0.9666	
P04406	GAPDH	Glyceraldehyde-3-phosphate dehydrogenase OS=Homo sapiens OX=9606 GI	36.053	24.8129	25.7692	-0.9563	
P10599	TXN	Thioredoxin OS=Homo sapiens OX=9606 GN=TXN PE=1 SV=3	11.737	24.211	25.14	-0.929	
J3KS45	TMCO1	Calcium load-activated calcium channel (Fragment) OS=Homo sapiens OX=9	16.58	25.5095	26.331	-0.8215	
G3V3D2	SPATA7	Spermatogenesis-associated protein 7 OS=Homo sapiens OX=9606 GN=SPA	4.7625	28.0015	28.8042	-0.8027	
P29508	SERPINB3	Serpin B3 OS=Homo sapiens OX=9606 GN=SERPINB3 PE=1 SV=2	44.564	24.3432	25.1255	-0.7823	
A8WI62	BRPF3	Bromodomain and PHD finger-containing protein 3 (Fragment) OS=Homo sapi	10.903	24.1384	23.3968	0.7416	
P63261	ACTG1	Actin, cytoplasmic 2 OS=Homo sapiens OX=9606 GN=ACTG1 PE=1 SV=1	41.792	20.6025	19.8985	0.704	
P05089	ARG1	Arginase-1 OS=Homo sapiens OX=9606 GN=ARG1 PE=1 SV=2	34.735	26.1526	25.5068	0.6458	
P12273	PIP	Prolactin-inducible protein OS=Homo sapiens OX=9606 GN=PIP PE=1 SV=1	16.572	23.0636	23.6948	-0.6312	
P07355	ANXA2	Annexin A2 OS=Homo sapiens OX=9606 GN=ANXA2 PE=1 SV=2	38.604	24.1569	24.783	-0.6261	
P42357	HAL	Histidine ammonia-lyase OS=Homo sapiens OX=9606 GN=HAL PE=1 SV=1	72.697	21.7933	22.4172	-0.6239	
M0QZU1	RPL13A	60S ribosomal protein L13a OS=Homo sapiens OX=9606 GN=RPL13A PE=1	4.7176	19.9188	19.364	0.5548	
F8WD96	CTSD	Cathepsin D OS=Homo sapiens OX=9606 GN=CTSD PE=1 SV=1	30	22.8908	22.389	0.5018	
Q5T2N8	ATAD3C	ATPase family AAA domain-containing protein 3C OS=Homo sapiens OX=960	46.379	21.969	22.4692	-0.5002	
Q6TDU7	CASC1	Protein CASC1 OS=Homo sapiens OX=9606 GN=CASC1 PE=1 SV=2	83.159	25.0867	25.5784	-0.4917	
P31025	LCN1	Lipocalin-1 OS=Homo sapiens OX=9606 GN=LCN1 PE=1 SV=1	19.25	21.4637	21.0331	0.4306	
Q6UWP8	SBSN	Suprabasin OS=Homo sapiens OX=9606 GN=SBSN PE=1 SV=2	60.54	24.4497	24.04	0.4097	
P17066	HSPA6	Heat shock 70 kDa protein 6 OS=Homo sapiens OX=9606 GN=HSPA6 PE=1	71.027	23.2521	22.8584	0.3937	
E5RK01	FAXDC2	Fatty acid hydroxylase domain-containing protein 2 (Fragment) OS=Homo sap	3.5811	27.2237	26.8483	0.3754	
H0YFI5	CRACR2A	EF-hand calcium-binding domain-containing protein 4B (Fragment) OS=Homo	13.867	22.9996	23.3735	-0.3739	
B1AN99	PRSS3	Trypsin-3 (Fragment) OS=Homo sapiens OX=9606 GN=PRSS3 PE=1 SV=8	19.288	26.2965	26.6603	-0.3638	
P22735	TGM1	Protein-glutamine gamma-glutamyltransferase K OS=Homo sapiens OX=9606	89.786	21.0449	21.3936	-0.3487	
Q5T749	KPRP	Keratinocyte proline-rich protein OS=Homo sapiens OX=9606 GN=KPRP PE=	64.135	23.3838	23.7123	-0.3285	
B0YJC4	VIM	Vimentin OS=Homo sapiens OX=9606 GN=VIM PE=1 SV=1	49.653	23.3795	23.0533	0.3262	
P14923	JUP	Junction plakoglobin OS=Homo sapiens OX=9606 GN=JUP PE=1 SV=3	81.744	26.1901	26.4946	-0.3045	
F8VY01	FGD6	FYVE, RhoGEF and PH domain-containing protein 6 OS=Homo sapiens OX=9	135.5	23.5384	23.2489	0.2895	
H0YFC6	RAN	GTP-binding nuclear protein Ran (Fragment) OS=Homo sapiens OX=9606 GN	11.568	22.5188	22.7893	-0.2705	
P05783	KRT18	Keratin, type I cytoskeletal 18 OS=Homo sapiens OX=9606 GN=KRT18 PE=1	48.057	28.4373	28.696	-0.2587	
Q5ST81	TUBB	Tubulin beta chain OS=Homo sapiens OX=9606 GN=TUBB PE=1 SV=1	41.742	23.1255	22.8807	0.2448	
Q5VTE0	EEF1A1P5	Putative elongation factor 1-alpha-like 3 OS=Homo sapiens OX=9606 GN=EE	50.184	24.1527	23.9154	0.2373	
F5GZQ4	LDHA	L-lactate dehydrogenase A chain (Fragment) OS=Homo sapiens OX=9606 GN	7.8752	20.7987	21.0313	-0.2326	

F8WBR5	CALM2	Calmodulin OS=Homo sapiens OX=9606 GN=CALM2 PE=1 SV=1	7.372	20.6322	20.8545	-0.2223
P32119	PRDX2	Peroxiredoxin-2 OS=Homo sapiens OX=9606 GN=PRDX2 PE=1 SV=5	21.892	24.7127	24.4936	0.2191
G3V1N2	HBA2	HCG1745306, isoform CRA_a OS=Homo sapiens OX=9606 GN=HBA2 PE=1	11.948	20.614	20.3954	0.2186
Q14152	EIF3A	Eukaryotic translation initiation factor 3 subunit A OS=Homo sapiens OX=9606	166.57	19.1342	19.3495	-0.2153
Q08188	TGM3	Protein-glutamine gamma-glutamyltransferase E OS=Homo sapiens OX=9606	76.631	24.7197	24.928	-0.2083
Q96P63	SERPINB12	Serpin B12 OS=Homo sapiens OX=9606 GN=SERPINB12 PE=1 SV=1	46.276	24.8407	25.0329	-0.1922
P31944	CASP14	Caspase-14 OS=Homo sapiens OX=9606 GN=CASP14 PE=1 SV=2	27.679	24.1949	24.3719	-0.177
P15924	DSP	Desmoplakin OS=Homo sapiens OX=9606 GN=DSP PE=1 SV=3	331.77	27.5098	27.6733	-0.1635
P01040	CSTA	Cystatin-A OS=Homo sapiens OX=9606 GN=CSTA PE=1 SV=1	11.006	26.3849	26.5452	-0.1603
P25311	AZGP1	Zinc-alpha-2-glycoprotein OS=Homo sapiens OX=9606 GN=AZGP1 PE=1 SV	34.258	24.5866	24.4364	0.1502
Q96FQ6	S100A16	Protein S100-A16 OS=Homo sapiens OX=9606 GN=S100A16 PE=1 SV=1	11.801	21.8845	21.7389	0.1456
Q16610	ECM1	Extracellular matrix protein 1 OS=Homo sapiens OX=9606 GN=ECM1 PE=1 S	60.673	21.371	21.4967	-0.1257
U3KQK0	H2BC15	Histone H2B OS=Homo sapiens OX=9606 GN=H2BC15 PE=1 SV=1	18.804	24.4324	24.3256	0.1068
Q08554	DSC1	Desmocollin-1 OS=Homo sapiens OX=9606 GN=DSC1 PE=1 SV=2	99.986	24.5468	24.4413	0.1055
P62805	H4C1	Histone H4 OS=Homo sapiens OX=9606 GN=H4C1 PE=1 SV=2	11.367	24.2886	24.1868	0.1018
F8WE04	HSPB1	Heat shock protein beta-1 OS=Homo sapiens OX=9606 GN=HSPB1 PE=1 SV	20.406	24.3161	24.2273	0.0888
Q5T750	XP32	Skin-specific protein 32 OS=Homo sapiens OX=9606 GN=XP32 PE=1 SV=1	26.238	23.9576	23.882	0.0756
P60709	ACTB	Actin, cytoplasmic 1 OS=Homo sapiens OX=9606 GN=ACTB PE=1 SV=1	41.736	28.3863	28.4301	-0.0438
F5H5D3	TUBA1C	Tubulin alpha chain OS=Homo sapiens OX=9606 GN=TUBA1C PE=1 SV=1	57.73	23.0639	23.1046	-0.0407
Q2L6G8	CDSN	Corneodesmosin OS=Homo sapiens OX=9606 GN=CDSN PE=1 SV=1	51.539	22.3658	22.3966	-0.0308
A0A0A0MSI0	PRDX1	Peroxiredoxin-1 (Fragment) OS=Homo sapiens OX=9606 GN=PRDX1 PE=1 S	18.976	22.047	22.0327	0.0143
Q02413	DSG1	Desmoglein-1 OS=Homo sapiens OX=9606 GN=DSG1 PE=1 SV=2	113.75	26.6714	26.6574	0.014
P63267	ACTG2	Actin, gamma-enteric smooth muscle OS=Homo sapiens OX=9606 GN=ACTG	41.876	23.5723	23.5829	-0.0106
P81605	DCD	Dermcidin OS=Homo sapiens OX=9606 GN=DCD PE=1 SV=2	11.284	27.9069	27.9126	-0.0057
P0CG48	UBC	Polyubiquitin-C OS=Homo sapiens OX=9606 GN=UBC PE=1 SV=3	77.038	29.8914	29.8941	-0.0027
