# Supplementary material for: Cullin‐associated and neddylation‐dissociated 1 regulate reprogramming of lipid metabolism through SKP1‐Cullin‐1‐F‐boxFBXO11‐mediated heterogeneous nuclear ribonucleoprotein A2/B1 ubiquitination and promote hepatocellular carcinoma
Source: Clin Transl Med. 2023 Oct 14;13(10):e1443. doi: 10.1002/ctm2.1443 (PMC10576442; doi:10.1002/ctm2.1443)
Supplement: Supplementary file 12 — Supporting Information [file CTM2-13-e1443-s004.pdf]

Supplementary Table. S4

Results of mass spectrometry following IP with an anti-CUL1 antibody in CAND1 knockdown cells and control cells.

Accession	Gene	shCAND1/shNC	log2 shCAND1/shNC	Diff Sig
P15924	DSP	0.037271759	-4.745773284	-
Q86VP6	CAND1	0.157507027	-2.666510108	+
P06702	S100A9	0.327447336	-1.610665202	-
A0A075B719	CEP152	0.610148294	-0.71276817	-
P81605	DCD	0.720698496	-0.472532261	
Q08554	DSC1	0.72585761	-0.46224153	
Q08188	TGM3	0.758095486	-0.39954852	
P47929	LGALS7	0.844363046	-0.244064655	
Q96P63	SERPINB12	0.848220023	-0.237489555	
Q02413	DSG1	0.890377723	-0.167510598	
A0A2R8Y5P9	SHROOM3	0.925124506	-0.112280554	
P14923	JUP	1.012907255	0.018502083	
P10599	TXN	1.019915012	0.02844894	
A0A2R8Y4E6	PDE8B	1.026609983	0.037888194	
Q8IV33	KIAA0825	1.085876651	0.11886023	
E5RGW4	NPM1	1.120396784	0.164009748	
P62805	H4C1	1.167695767	0.223664441	
H0YNB8	ANXA2	1.333803195	0.41554581	
Q9H2T7	RANBP17	1.342984052	0.425442173	
P01042	KNG1	1.404185816	0.48973386	
Q96EF6	FBXO17	1.504274501	0.589067855	+
O94952	FBXO21	1.539944747	0.622878588	+
Q9UKT8	FBXW2	1.579622633	0.659579944	+
Q8NEZ5	FBXO22	1.698027253	0.763859614	+
H3BV27	RPS15A	1.701892112	0.767139583	+
P15924	DSP	1.717388136	0.78021613	+
E9PCS6	LAMB1	1.740838271	0.799782178	+
H0YFI5	CRACR2A	1.776601679	0.829120259	+
P63208	SKP1	1.802278074	0.849821622	+
Q13309	SKP2	1.824138363	0.867215164	+
H0Y9N7	IBTK	1.847621497	0.885669237	+
Q96QH2	PRAM1	1.97093849	0.978882753	+
F5H5D3	TUBA1C	2.075329991	1.053340753	+
Q8NCQ5	FBX15	2.125209728	1.087605221	+
Q53SF7	COBLL1	2.130210913	1.090996279	+
Q99880	H2BC13	2.174258395	1.120523404	+
Q969H0	FBW7	2.356329082	1.236541038	+
J3KRB3	RPL17	2.562064008	1.357306519	+
Q9H4M3	FBXO44	2.583446529	1.369297024	+
Q8IXT1	DDIAS	2.599334638	1.378142378	+
Q9UKT7	FBXL3	2.740973994	1.45468864	+
P0DO92	CDIPTOSP	2.853177243	1.512569372	+

Q9NXK8	FBXL12	2.89025025	1.531194413	+
Q96KN9	GJD4	2.917501508	1.544733402	+
Q9UKT4	FBXO5	2.967519486	1.569257503	+
P60709	ACTB	2.996339829	1.583201256	+
Q96CD0	FBXL8	3.213376038	1.684089819	+
P31512	FMO4	3.712942181	1.892562849	+
K7EII2	CEP76	3.793035544	1.923352891	+
E9PI65	HSPA8	3.88430598	1.957656851	+
Q86XK2	FBXO11	4.377906431	2.13024112	+
Q5T2N8	ATAD3C	5.933655058	2.568921061	+
Q08380	LGALS3BP	6.287699289	2.652532222	+
F8WE04	HSPB1	8.475487515	3.083296355	+
P38646	HSPA9	32.08994531	5.004049426	+
Q01082	SPTBN1	57.9568216	5.856906573	+
