# Supplementary material for: Cullin‐associated and neddylation‐dissociated 1 regulate reprogramming of lipid metabolism through SKP1‐Cullin‐1‐F‐boxFBXO11‐mediated heterogeneous nuclear ribonucleoprotein A2/B1 ubiquitination and promote hepatocellular carcinoma
Source: Clin Transl Med. 2023 Oct 14;13(10):e1443. doi: 10.1002/ctm2.1443 (PMC10576442; doi:10.1002/ctm2.1443)
Supplement: Supplementary file 13 — Supporting Information [file CTM2-13-e1443-s010.pdf]

Supplementary Table. S5

Results of MS following IP of FBXO11 and identified hnRNPA2B1 in the FBXO11 co-precipitation.

Accession	Gene	Mw(kDa)	Length	Peptides
P15924	DSP	331.774	2871	9
A0A0A0MS07	IGHG1	31.983	295	1
P60709	ACTB	41.737	375	2
A0A590UJC4	CUL1	78.37	1049	3
E9PCS6	LAMB1	10.082	91	1
P01042	KNG1	71.957	644	1
Q53SF7	COBLL1	123.868	1128	1
A0A2R8Y5P9	SHROOM3	208.05	1915	1
P22626	HNRNPA2B1	37.43	353	2
P62805	H4C1	11.367	103	2
Q99880	H2BC13	13.952	126	2
F5H5D3	TUBA1C	57.73	519	2
P06702	S100A9	13.242	114	1
P10599	TXN	11.738	105	2
P14923	JUP	81.745	745	2
P38646	HSPA9	73.681	679	1
P81605	DCD	11.284	110	2
Q08554	DSC1	99.987	894	2
Q5T2N8	ATAD3C	46.38	411	1
Q8IV33	KIAA0825	147.735	1275	1
Q96P63	SERPINB12	46.276	405	2
Q96QH2	PRAM1	73.969	670	1
A0A2U3TZL8	KIF23	92.683	810	1
C9JB04	WIPF1	15.08	157	1
E5RGW4	NPM1	6.863	59	1
E9PI65	HSPA8	17.916	168	1
F8WE04	HSPB1	20.406	186	1
H0YFI5	CRACR2A	13.978	118	1
H0YNB8	ANXA2	5.125	47	1
K7EII2	CEP76	8.577	74	1
M0QXB0	LSM4	7.83	69	1
P31512	FMO4	63.343	558	1
P31944	CASP14	27.68	242	1
P47929	LGALS7	15.075	136	1
Q08188	TGM3	76.632	693	1
Q8IXT1	DDIAS	111.616	998	1
Q9H2T7	RANBP17	124.375	1088	1
Q9NR99	MXRA5	312.15	2828	1
F8W881	FAM120C	99.352	895	1
P0DO92	CDIPTOSP	9.002	83	1
